# LncRNA TUG1 alleviates cardiac hypertrophy by targeting miR‐34a/DKK1/Wnt‐β‐catenin signalling

**DOI:** 10.1111/jcmm.15067

**Published:** 2020-02-14

**Authors:** Qingxia Fang, Ting Liu, Chenhuan Yu, Xiuli Yang, Yanfei Shao, Jiana Shi, Xiaolan Ye, Xiaochun Zheng, Jieping Yan, Danfeng Xu, Xiaozhou Zou

**Affiliations:** ^1^ Department of Pharmacy Zhejiang Provincial People's Hospital Hangzhou China; ^2^ People's Hospital of Hangzhou Medical College Hangzhou China; ^3^ Department of pharmacy Affiliated Hangzhou First People's Hospital Zhejiang University School of Medicine Hangzhou China; ^4^ Key Laboratory of Experimental Animal and Safety Evaluation Zhejiang Academy of Medical Sciences Hangzhou China

**Keywords:** cardiac hypertrophy, DKK 1, lncRNA TUG1, miR‐34a, Wnt/β‐catenin signalling

## Abstract

The current study was designed to explore the role and underlying mechanism of lncRNA taurine up‐regulated gene 1 (TUG1) in cardiac hypertrophy. Mice were treated by transverse aortic constriction (TAC) surgery to induce cardiac hypertrophy, and cardiomyocytes were treated by phenylephrine (PE) to induce hypertrophic phenotype. Haematoxylin‐eosin (HE), wheat germ agglutinin (WGA) and immunofluorescence (IF) were used to examine morphological alterations. Real‐time PCR, Western blots and IF staining were used to detect the expression of RNAs and proteins. Luciferase assay and RNA pull‐down assay were used to verify the interaction. It is revealed that TUG1 was up‐regulated in the hearts of mice treated by TAC surgery and in PE‐induced cardiomyocytes. Functionally, overexpression of TUG1 alleviated cardiac hypertrophy both in vivo and in vitro. Mechanically, TUG1 sponged and sequestered miR‐34a to increase the Dickkopf 1 (DKK1) level, which eventually inhibited the activation of Wnt/β‐catenin signalling. In conclusion, the current study reported the protective role and regulatory mechanism of TUG1 in cardiac hypertrophy and suggested that TUG1 may serve as a novel molecular target for treating cardiac hypertrophy.

## 1 INTRODUCTION

Cardiac hypertrophy, classified as physiological and pathological hypertrophy, is an adaptive response of the heart to keep normal cardiac function in the condition of pathological injury or abnormal stress.^1^ Physiological cardiac hypertrophy, caused by pregnancy or sports training, has normal morphological characteristics and helpful influences on the heart.^2^ Pathological cardiac hypertrophy, accompanied by maladaptive cardiac remodelling, altered cardiac morphology as well as abnormal cardiac gene expressions, is the key induction factor for heart failure progression.^3^ Although many factors have been confirmed to induce cardiac hypertrophy,^4^ the underlying molecular mechanisms have not been stated clearly.

LncRNAs, a series of highly conserved non‐coding RNAs, are composed of more than 200 nucleotides.^5^, ^6^ With continuous advances in sequencing technology and large‐scale genome sequencing projects, lncRNAs have become a major focus of research. LncRNAs usually exert their functions via recruiting specific RNA‐binding proteins, promoting target gene expression, influencing angiogenesis and by functioning as a competing endogenous RNA (ceRNA).^5^, ^6^ Whole‐genome transcriptome analysis showed that many cardiac‐specific lncRNAs play critical regulating roles in hypertrophic response and cardiac remodelling, indicating that lncRNAs may be the significant biological markers and therapeutic targets for cardiac hypertrophy 7, 8

LncRNA taurine up‐regulated gene 1 (TUG1) is located in chromosome 22p12 and identified as a transcript up‐regulated via taurine.^9^ It is originally found in taurine‐treated mouse retinal cells, and inhibition of TUG1 causes malformed outer segments of transfected photoreceptors through increasing apoptosis in the newborn retina.^9^ TUG1 is ubiquitously expressed and has been documented to regulate different tumour development and cell metabolism through influencing cell proliferation, invasion, metastasis, apoptosis, differentiation and drug resistance.^10^ Recently, TUG1 is also reported to exert a crucial regulation in the processes of cardiovascular diseases. TUG1 is increased during cardiac fibroblast‐myofibroblast transformation (FMT) and inhibition of TUG1 ameliorates FMT through sponging miR‐29c.^11^ TUG1 contributes to cerebral ischaemia/reperfusion injury by sponging miR‐145 to up‐regulate AQP4 expression.^12^ Knockdown of TUG1 reduces hypoxia‐induced pulmonary smooth muscle cells proliferation and survival through sponging miR‐328. Also, knockdown of TUG1 attenuates pulmonary vascular remodelling in hypoxic pulmonary hypertension through the Foxc1‐mediated NOTCH signalling pathway.^13^ TUG1 inhibition ameliorates atherosclerosis by modulating FGF1 via sponging miR‐133a.^14^ However, the role of TUG1 in cardiac hypertrophy has not yet been stated clearly. We, therefore, wandered to explore the role and underlying mechanism of TUG1 in cardiac hypertrophy.

## 2 MATERIALS AND METHODS

### 2.1 Animal experiments

C57BL/6 mice (male, 8 weeks old) were sourced from the Key Laboratory of Experimental Animal and Safety Evaluation, Zhejiang Academy of Medical Sciences. All the experimental procedures were performed by and approved by the Animal Ethics Committee of Zhejiang Provincial People's Hospital. Mice underwent a TAC surgery to induce the cardiac pressure overload models as described in a previous study.^15^ Moreover, mice were injected with rAAV9 (4 × 10^11^ vector genomes (vg)/mouse) carrying an empty vector or TUG1 via the tail vein. Real‐time quantitative assay was used to quantify the TUG1 copy number. DNeasy Tissue Kits (Qiagen) was used to extract genomic AAV9‐TUG1 vector DNA from the frozen cardiac tissues. To examine vector genome copies, 100 ng of each sample was applied in duplicate. The copy number standard range of plasmid DNA was 10‐10^8^ copies/µL. 150 copies/µL DNA was the lower limit detection. The full description of the establishment of animal models is available in the online data supplements.

### 2.2 Echocardiography and haemodynamic detection

After being anaesthetized and placed on a platform, mice underwent echocardiography via the ultrasound machine Vevo2100 high‐resolution imaging system (VisualSonics). The instantaneous intraventricular pressure and volume were measured via inserting a 1.4‐F pressure‐volume catheter (SPR835, Millar Instruments) into the left ventricle of mice, as described previously.^15^

### 2.3 Cell isolation and culture

As described in previous,^16^, ^17^ cardiomyocytes and cardiac fibroblasts were isolated from neonatal mice (1‐2 days) and adult mice (male, 8 weeks old). The full description of cell culture and procedure are available in the online data supplements.

### 2.4 Cell transfection

Cells were plated in a 24‐well cell culture plate. Then, TUG1 siRNA, miR‐34a mimic, miR‐34a inhibitor, DKK1 siRNA and the negative control were respectively transfected into cells by lipofectamine 2000. The full description of the cell transfection procedure is available in the online data supplements.

### 2.5 Real‐time PCR analysis

Trizol reagent (Invitrogen) was used to extract total RNA from cardiomyocytes and cardiac tissues. After total RNA extracting, the Prime Script reverse transcription reagent Kit (CWBIO) was used to produce cDNA. An ABI 7300 real‐time PCR system was used to perform a Real‐time PCR process. The full description of Real‐time PCR procedure is available in the online data supplements and Table S1.

### 2.6 Cell immunostaining analysis

After fixation, cells were treated with 0.5% Triton‐X 100, α‐actinin primary antibody (Table S2) and fluorescence‐conjugated secondary antibody to conduct immun ostaining. The full description of the cell immunostaining procedure is available in the online data supplements and Table S2.

### 2.7 Protein/DNA ratio detection

As a previous study described,^18^ after treatment, cardiomyocytes were washed with PBS. Then, the cells were incubated with perchloric acid and collected by centrifugation (10 000 × *g*, 10 min). After centrifugation, the precipitates were treated with KOH for 30 min at 70°C. Lowry method Hoechst dye 33 258 was used to examine protein and DNA contents.

### 2.8 Western blot examination

Protein from frozen tissues and cells was extracted. After denaturation, proteins were separated by SDS‐PAGE and transferred to polyvinylidene fluoride membrane. After blocking, the membranes were incubated with primary antibodies and secondary antibodies. Enhanced chemiluminescence was used to visualize the target proteins. The full description of the Western blot procedure is available in the online data supplements and Table S3.

### 2.9 Histological analysis

Heart tissues were fixed, embedded in paraffin and sectioned. The morphology was detected by H&E or WGA staining. The full description of histological analysis and procedure are available in the online data supplements.

### 2.10 RNA‐binding protein immunoprecipitation assay (RIP assay)

As described previously,^19^ Magna RIP Kit (EMD Millipore) was used to carry out RNA‐binding protein immunoprecipitation (RIP) assay. Using protein G Sepharose beads, anti‐Ago2 antibody (Abnova) or immunoglobulin G (IgG; Santa Cruz Biotechnology) was used to immune‐precipitate lysed cell extracts. After gradient elution, trizol was used to extract bound RNA. The results were quantified by real‐time PCR as described previously.

### 2.11 Luciferase reporter assay

The amplified sequence of TUG1 or DKK1 including the predicted miR‐34a binding sequence was inserted into a pmirGLO Dual‐luciferase Target Expression Vector (Promega) to produce Wt‐TUG1 or Wt‐TUG1 reporter vector. Dual‐Luciferase Reporter Assay System (Promega) was used to carry out Luciferase reporter assay. The full description of luciferase reporter assay is available in the online data supplements.

### 2.12 RNA pull‐down assay

50 nM biotinylated‐miR‐34a(bio‐miR‐34a; ThermoFisher Scientific, Ltd.) were transfected into Cardiomyocytes. After incubating for 48 hours, the cells were collected and lysed. The lysate was then incubated with beads (S3762; Sigma‐Aldrich Chemical Company) and pre‐coated with RNase‐free BSA and yeast tRNA at 4°C overnight. Subsequently, the cells were washed twice with pre‐cooled lysis buffer, three times with low‐salt buffer and once with high‐salt buffer. Finally, the bound RNA was purified by Trizol, and Real‐time PCR was conducted to detect the enrichment of lncRNA TUG1.

### 2.13 Statistical analysis

Data were presented as mean ± SEM Double‐sided Student's t test was used for the comparisons between groups. Statistical analysis was performed by GraphPad Prism (Version 6.0). *P* < .05 was considered to indicate a statistically different.

## 3 RESULTS

### 3.1 TUG1 was induced in hypertrophic hearts

**Figure 1:**
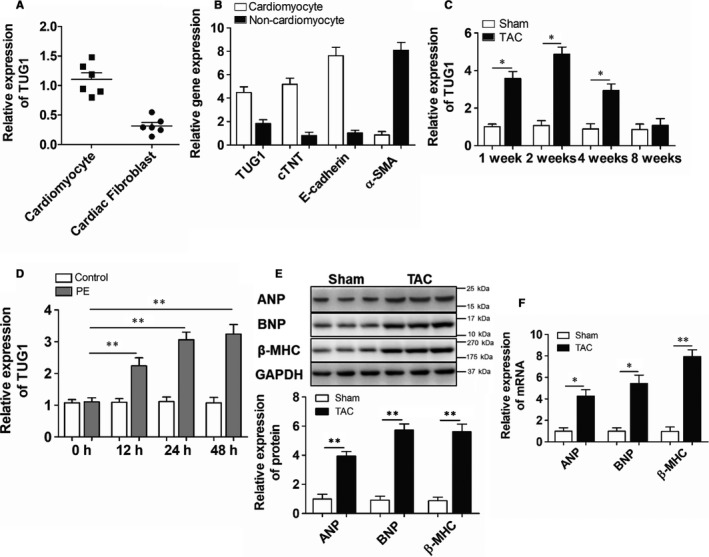
TUG1 expression was up‐regulated in hypertrophic hearts. A, Real‐time PCR analyses of TUG1 expression in isolated neonatal mice cardiomyocytes and cardiac fibroblasts. (n = 6). B, Real‐time PCR analyses of the expressions of TUG1, cTNT, E‐cadherin, and α‐SMA in isolated cardiomyocytes and non‐cardiomyocytes from adult mouse hearts. (n = 3). C, Real‐time PCR analyses of TUG1 expression in TAC‐treated hearts of mice for 1, 2, 4 and 8 wk. (n = 6; *P < .01 vs Sham group). D, Real‐time PCR analyses of TUG1 expression in isolated neonatal mouse cardiomyocytes treated with PE for 0, 12, 24 and 48 h. (n = 3; **P < .01 vs Control group). E, Western blot and Real‐time PCR analyses for the levels of hypertrophic biomarkers in TAC‐treated hearts for 2 wk. (n = 6; *P < .05 vs Sham group, **P < .01 vs Sham group). F, Real‐time PCR analyses for the levels of hypertrophic biomarkers in TAC‐treated hearts for 2 wk. (n = 6; *P < .05 vs Sham group, **P < .01 vs Sham group)

The expression of TUG1 in different cell types was detected. As shown in Figure 1A, TUG1 was abundantly expressed in neonatal mouse cardiomyocytes. Also, TUG1 was expressed predominantly in adult mouse cardiomyocytes which were identified by cardiomyocyte markers (cardiomyocytes cardiac troponin T (cTNT) and E‐cadherin; Figure 1B). We next examined the expression of TUG1 in hypertrophic hearts. As shown in Figure 1C, TUG1 expression was increased in the hearts of mice treated by TAC from 1 to 2 weeks, while, this up‐regulation declined after TAC treating for 4 weeks and returned to control levels by 8 weeks after TAC surgery. In PE‐treated cardiomyocytes, TUG1 expression was also increased gradually and reached its apex at 48 hours (Figure 1D). Moreover, the protein and mRNA levels of hypertrophic biomarkers including atrial natriuretic peptide (ANP), brain natriuretic peptide (BNP) and β‐myosin heavy chain (β‐MHC) were increased in the hypertrophic hearts induced by TAC surgery (Figure 1E,F). These results demonstrated that TUG1 was enriched in cardiomyocytes and was induced during cardiac hypertrophy.

### 3.2 TUG1 inhibition induced cardiomyocyte hypertrophy and TUG1 overexpression alleviated cardiomyocyte hypertrophy

**Figure 2:**
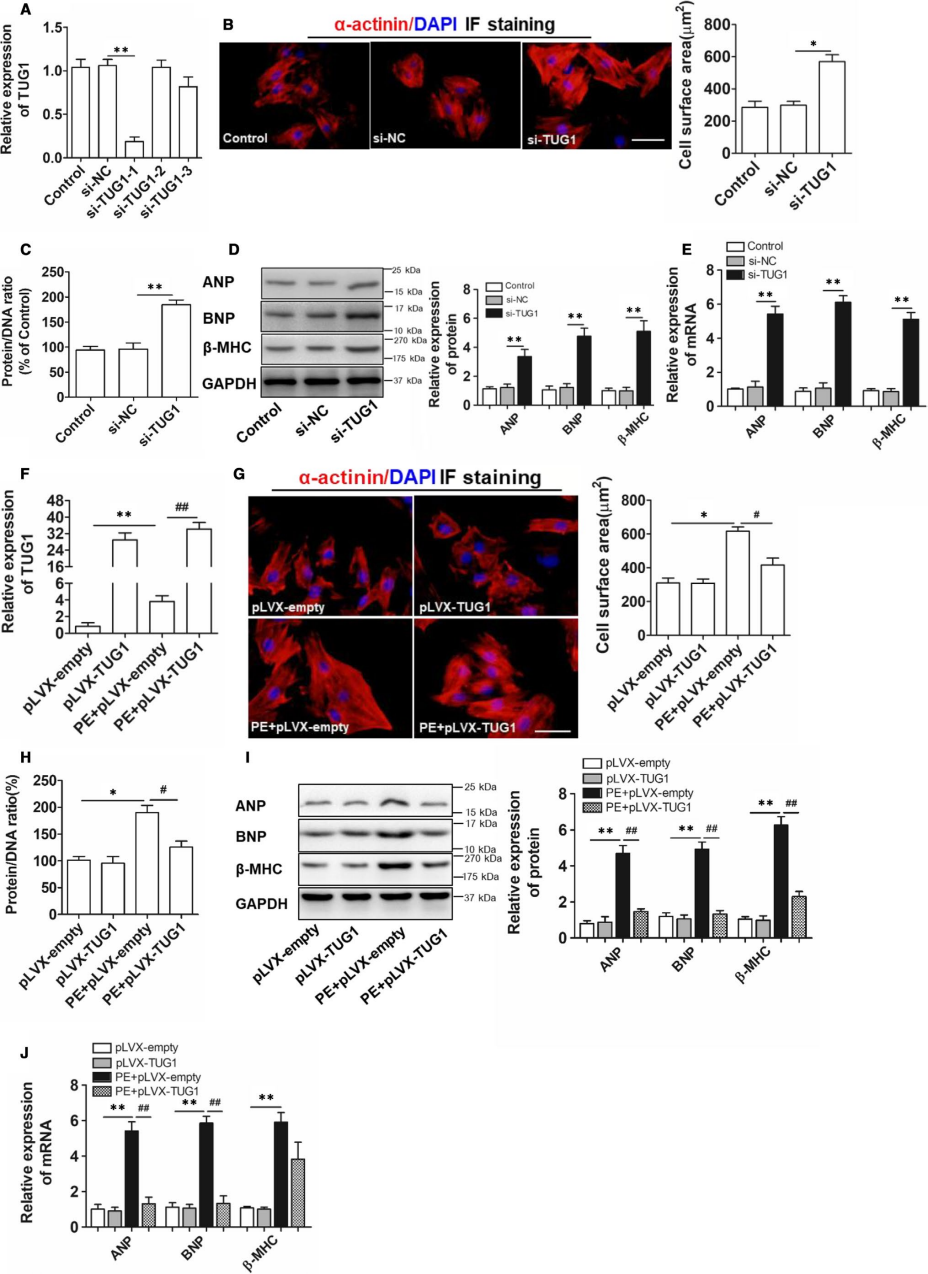
Inhibition of TUG1 induced a hypertrophic response and overexpression of TUG1 attenuated the hypertrophic response in PE‐treated cardiomyocytes. A, Real‐time PCR analyses for interference efficiency of si‐TUG1 in cardiomyocytes. (n = 3; **P < .01 vs si‐NC group). B, Immunofluorescence staining analyses for cardiomyocytes treated with si‐TUG1 and the quantified cell area results. Both cell groups were stained with antibodies directed against α‐actinin and with DAPI for nuclear staining. (bar = 25 μm, *P < .05 vs si‐NC group). C, Protein/DNA ratio examination for cardiomyocytes treated with si‐TUG1. (n = 3; **P < .01 vs si‐NC group). D, Western blot analyses for the levels of hypertrophic biomarkers in cardiomyocytes treated with si‐TUG1. (n = 3; **P < .01 vs si‐NC group). E, Real‐time PCR analyses for the relative mRNA levels of hypertrophic biomarkers in cardiomyocytes treated with si‐TUG1. (n = 3; **P < .01 vs si‐NC group). F, Real‐time PCR examination for TUG1 expression in cardiomyocytes treated with pLVX‐TUG1 before PE treatment. (n = 3; **P < .01 vs pLVX‐empty group; ##*P* < .01 vs PE + pLVX‐empty group). G, Immunofluorescence staining analyses for cardiomyocytes treated with pLVX‐TUG1 before PE treatment and the quantified cell area results. Both cell groups were stained with antibodies directed against α‐actinin and with DAPI for nuclear staining. (bar = 25 μm, *P < .05 vs pLVX‐empty group; #*P* < .05 vs PE + pLVX‐empty group). H, Protein/DNA ratio examination for cardiomyocytes treated with pLVX‐TUG1 before PE treatment. (n = 3; *P < .05 vs pLVX‐empty group; #*P* < .05 vs PE + pLVX‐empty group). I, Western blot examination for the protein levels of hypertrophic markers in cardiomyocytes treated with pLVX‐TUG1 before PE treatment. (n = 3; **P < .01 vs pLVX‐empty group; ##*P* < .01 vs PE + pLVX‐empty group). J, Real‐time PCR examination for the relative mRNA levels of hypertrophic biomarkers in cardiomyocytes treated with pLVX‐TUG1 before PE induction. (n = 3; **P < .01 vs pLVX‐empty group; ##*P* < .01 vs PE + pLVX‐empty group)

To explore the role of TUG1 in cardiomyocyte hypertrophy, si‐TUG1 and pLVX‐TUG1 were used to inhibit and overexpress TUG1 respectively. The expression of TUG1 was suppressed in cardiomyocytes by si‐TUG1 (Figure 2A). TUG1 inhibition induced cardiomyocyte hypertrophy, manifested by the increased cell surface area (Figure 2B), up‐regulated protein/DNA ratio (Figure 2C) and enhanced expression of hypertrophic biomarkers (Figure 2D,E). Moreover, pLVX‐TUG1 significantly increased the expression of TUG1 (Figure S1A,B and Figure 2F), and partially suppressed cardiomyocyte hypertrophy induced by PE, revealed by the decreased cell surface area (Figure 2G), reduced protein/DNA ratio (Figure 2H) and down‐regulated levels of hypertrophic biomarkers (Figure 2I,J). Besides, TUG1 overexpression did not affect the normal cardiomyocytes (Figure 2G‐J). These results demonstrated that TUG1 was a critical regulator to counteract PE‐induced cardiomyocyte hypertrophy.

### 3.3 TUG1 targeted miR‐34a to regulate cardiomyocyte hypertrophy

**Figure 3:**
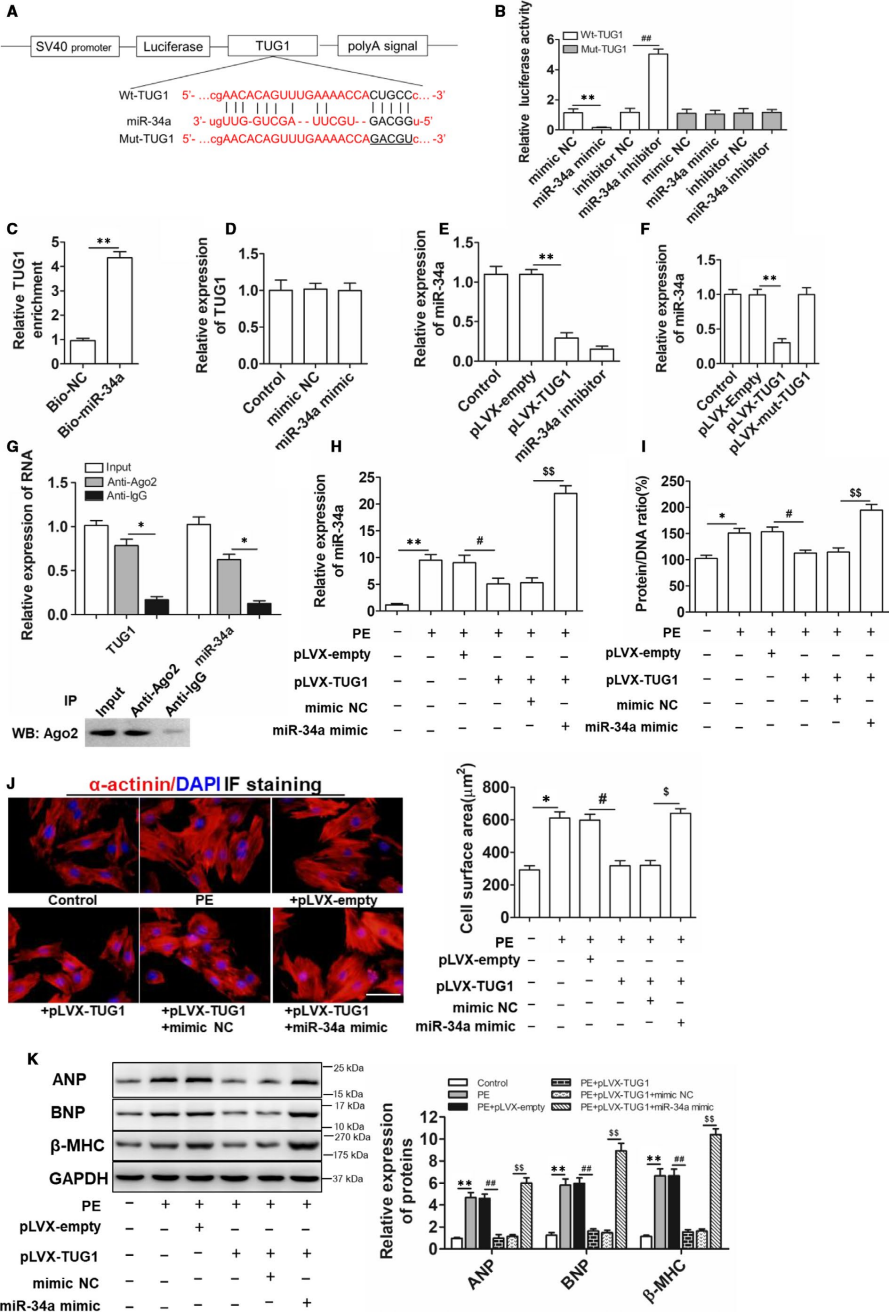
TUG1 regulated the hypertrophic response of cardiomyocytes by targeting miR‐34a. A, Putative binding sites (Wt) of TUG1 and miR‐34a, together with mutant sites of TUG1 in Mut‐TUG1 luciferase reporter. B, Luciferase activity analyses in cardiomyocytes co‐transfected with miR‐34a mimic, miR‐34a inhibitor and luciferase reporters containing Wt‐TUG1 or Mut 3'‐UTR. (n = 3; **P < .01 vs mimic Negative Control [mimic NC] group; ##*P* < .01 vs inhibitor Negative Control [inhibitor NC] group). C, The interaction between TUG1 and miR‐34a analysed by RNA pull‐down assay. (n = 4, **P < .01 vs Bio‐NC group). D, Real‐time PCR analyses for the expression of TUG1 after miR‐34a mimic transfection in cardiomyocytes. (n = 3). E, Real‐time PCR analyses for miR‐34a levels after overexpression of TUG1 in cardiomyocytes. (n = 3; **P < .01 vs pLVX‐empty group). F, Real‐time PCR analyses for the expression of miR‐34a in cardiomyocytes transfected with pLVX‐TUG1, or pLVX‐mut‐TUG1 (TUG1 overexpression plasmids containing the mutagenized miR‐34 binding site). (n = 3; **P < .01 vs pLVX‐empty group). G, Cell lysate incubated with an anti‐Ago2 antibody for RIP, and the TUG1 content detected by Real‐time PCR. (n = 3; *P < .05 vs Anti‐lgG group). H, Real‐time PCR examination for miR‐34a expression in cardiomyocytes transfected with pLVX‐ TUG1, or pLVX‐TUG1 + miR‐34a mimic prior to PE treatment.(n = 3; **P < .01 vs Control group; #*P* < .05 vs PE + pLVX‐empty group; $$*P* < .01 vs PE + pLVX‐TUG1 + mimic NC group). I, Protein/DNA ratio examination for cardiomyocytes treated with pLVX‐TUG1, or pLVX‐TUG1 + miR‐34a mimic prior to PE induction. (n = 3; *P < .05 vs Control group; #*P* < .05 vs PE + pLVX‐empty group; $$*P* < .01 vs PE + pLVX‐TUG1 + mimic NC group). J, Immunofluorescence staining analyses of cardiomyocytes treated with pLVX‐TUG1, or pLVX‐TUG1 + miR‐34a mimic before PE induction and the quantified cell area results. Both cell groups were stained with antibodies directed against α‐actinin and with DAPI for nuclear staining. (bar = 25 μm, *P < .05 vs Control group; #*P* < .05 vs PE + pLVX‐empty group; $*P* < .05 vs PE + pLVX‐TUG1 + mimic NC group). K, Western blot analyses for the levels of hypertrophic markers in cardiomyocytes treated with pLVX‐TUG1, or pLVX‐TUG1 + miR‐34a mimic prior to PE induction. (n = 3; **P < .01 vs Control group; ##*P* < .01 vs PE + pLVX‐empty group; $$*P* < .01 vs PE + pLVX‐TUG1 + mimic NC group)

Recently, the ceRNA mechanism of lncRNAs has attracted a lot of research interest. The concept of ceRNAs demonstrates that the RNA molecules that contain the binding sites (sequence complementarity) to a particular miRNA can competitively bind to this individual miRNA to reduce its functional availability.^20^ It is just like a function of sponge. LncRNAs can often act as ceRNAs, sponging and sequestering miRNAs, indirectly regulating the target genes of miRNAs, or specifically, releasing the target genes from repression by the targeted miRNA.^21^ Bioinformatics tools (miRDB, starBase) were used to analyse the miRNAs which can be targeted by TUG1. miR‐34a, sharing a highly conserved binding site with TUG1 (Figure 3A), regulates cardiac dysfunction in hypertrophic cardiomyopathy.^22^ After confirming the transfection efficiency of miR‐34a mimic and inhibitor in cardiomyocytes (Figure S1C,D), the dual‐luciferase reporter assay was performed. As shown in Figure 3B, miR‐34a mimic inhibited the luciferase activity of pmirGLO‐wild‐TUG1 (Wt‐TUG1) while miR‐34a inhibitor up‐regulated the luciferase activity as expected. Further, RNA pull‐down assay manifested that bio‐miR‐34a could enrich TUG1 in cardiomyocytes more than bio‐NC control probe, indicating the physical interaction between TUG1 and miR‐34a (Figure 3C). Besides, miR‐34a mimic did not affect the expression of TUG1 (Figure 3D). Moreover, overexpression of TUG1 inhibited the expression of miR‐34a (Figure 3E) and TUG1‐overexpressed plasmid with the mutagenized miR‐34 binding site did not regulate the expression of miR‐34a (Figure 3F), confirming a sponge effect of TUG1 on miR‐34. Further, Ago2 pull‐down analysis showed that both TUG1 and miR‐34a were detected in Ago2 pellet (Figure 3G), and high expression of miR‐34 and TUG1 in Ago2 pellets means the possible combination between them. The above results revealed TUG1 was a good sponge for miR‐34a. Moreover, we inferred in the presence of TUG1 excess, the repression of miR‐34 on other known miR‐34 targets should be at least partially released. Therefore, the expression of other known miR‐34a target genes including Sirt1, ZEB1 and Syntaxin 1A in cardiomyocytes treated by miR‐34a mimic or/and pLVX‐TUG1 was examined. As shown in Figure S1E‐G, miR‐34a overexpression inhibited the mRNA expression of these target genes, and TUG1 overexpression partially released this process. The above results confirmed TUG1 sponged and sequestered miR‐34a through a functional binding site but miR‐34a did not regulate TUG1 expression. Next, we examined the role of TUG1/miR‐34a signalling in cardiomyocyte hypertrophy. Cardiomyocytes were transfected with pLVX‐TUG1 and/or miR‐34a mimic before PE treatment. As shown in Figure 3H, miR‐34a up‐regulation induced by PE was repressed by TUG1 overexpression. More importantly, overexpression of miR‐34a partially weakened the protective role of TUG1 on PE‐induced cardiomyocyte hypertrophy, manifested by the higher protein/DNA ratio (Figure 3I), increased cell surface area (Figure 3J) and up‐regulation of hypertrophic biomarkers (Figure 3K). These findings suggested TUG1 alleviated PE‐induced cardiomyocyte hypertrophy through targeting miR‐34a.

### 3.4 DKK1 is the downstream target gene of miR‐34a and conveyed the hypertrophic response through the Wnt/β‐catenin signalling

**Figure 4:**
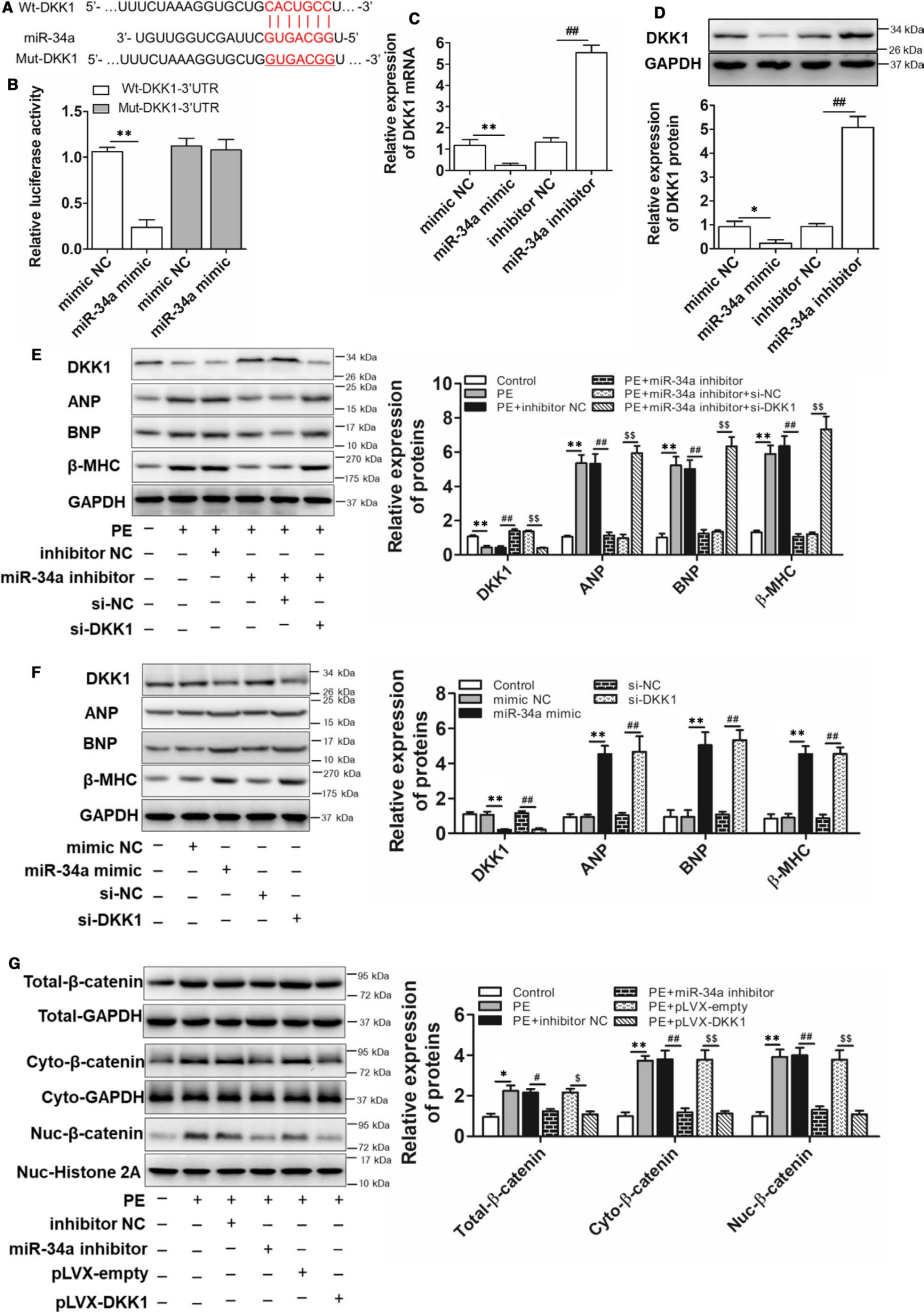
miR‐34a regulated cardiomyocytes hypertrophy through targeting DKK1 expression and activating canonical Wnt/β‐catenin signalling. A, Putative binding sites between DKK1 3'UTR and miR‐34a, along with mutant sites in Mut‐DKK1‐3'UTR reporter. B, Luciferase activity analyses in cardiomyocytes co‐transfected with miR‐34a mimic and luciferase reporters containing Wt‐DKK1 or Mut‐DKK1. (n = 3; **P < .01 vs mimic NC group). C, Real‐time PCR examination for the mRNA levels of DKK1 in cardiomyocytes co‐transfected with miR‐34a mimic or miR‐34a inhibitor. (n = 3; **P < .01 vs mimic NC group; ##*P* < .01 vs inhibitor NC group). D, Western blot analyses for the protein levels of DKK1 in cardiomyocytes co‐transfected with miR‐34a mimic or miR‐34a inhibitor. (n = 3; *P < .05 vs mimic NC group; ##*P* < .01 vs inhibitor NC group). E, Western blot analyses for the expressions of DKK1 and hypertrophic markers in cardiomyocytes transfected with miR‐34a inhibitor alone or together with si‐DKK1 prior to PE treatment. (n = 3; **P < .01 vs Control group; ##*P* < .01 vs PE + inhibitor NC group; $$*P* < .01 vs PE + miR‐34a inhibitor + si‐NC group). F, Western blot analyses for the expressions of DKK1 and hypertrophic markers in cardiomyocytes transfected with miR‐34a mimic alone or si‐DKK1 alone. (n = 3; **P < .01 vs mimic NC group; ##*P* < .01 vs si‐NC group). G, Western blot analyses for the activation of the Wnt/β‐catenin signalling in cardiomyocytes transfected with miR‐34a inhibitor or pLVX‐DKK1 before PE induction. (n = 3; *P < .05 vs Control group; **P < .01 vs Control group; #*P* < .05 vs PE + inhibitor NC group; ##*P* < .01 vs PE + inhibitor NC group; $*P* < .05 vs PE + pLVX‐empty group; $$*P* < .01 vs PE + pLVX‐empty group)

MiRNAs themselves are not hypertrophic executioners, and they always exert their effect through targeting hypertrophic regulating genes. Bioinformatics analysis was used to detect the target gene of miR‐34a. It is revealed miR‐34a shares a highly conversed binding site with DKK1 (Figure 4A). DKK1, acting as a potent Wnt signalling antagonist, binds to LRP5/6 and blocks the Fz‐LRP association, subsequently inducing specific inhibition of the Wnt/β‐catenin signalling pathway.^23^ Wnt/β‐catenin signalling has been reported to be an inducer for cardiac hypertrophy, and inactivation of this signalling ameliorated hypertensive heart disease.^24^ It is reported Dkk1 inhibits Wnt signalling to regulate early myocardial proliferation^25^ Dkk1 exacerbates doxorubicin‐induced cardiotoxicity by inhibiting the Wnt/β‐catenin signalling pathway.^26^ It is indicating a significant role of DKK1 in the heart diseases. We, therefore, examined if miR‐34a targets DKK1 to regulate cardiomyocyte hypertrophy. As shown in Figure 4B, miR‐34a mimic decreased the luciferase activity of pmirGLO‐wild‐DKK1 (Wt‐DKK1) but did not affect pmirGLO‐mut‐DKK1 (Mut‐DKK1). Besides, miR‐34a mimics suppressed DKK1 mRNA and miR‐34a inhibitor exerted opposite function (Figure 4C,D). It is indicating a targeted regulation of miR‐34a on DKK1 expression. Further, the role of miR‐34a/DKK1 signalling on cardiomyocyte hypertrophy was explored. Firstly, the transfection efficiency of DKK1 siRNA and pLVX‐DKK1 was confirmed in Figure S2A,B. Further, as shown in Figure 4E, inhibition of miR‐34a blocked DKK1 down‐regulation and alleviated cardiomyocyte hypertrophy induced by PE, and the process was cancelled by si‐DKK1. MiR‐34a mimic alone could also induce cardiomyocytes hypertrophy, which played a similar role with si‐DKK1 (Figure 4F). These results suggested that miR‐34a contributed to cardiac hypertrophy by targeting DKK1. As DKK‐1 is reported to regulate many disease processes through inhibiting the activation of Wnt/β‐catenin signalling, we next explored the activation of Wnt/β‐catenin signalling and regulated role of miR‐34a and DKK1 on this signalling. As shown in Figure 4G, PE‐induced β‐catenin accumulation both in the cell cytoplasm and nuclear of cardiomyocytes, and the process was alleviated by either miR‐34a inhibition or DKK1 overexpression. These results demonstrated that miR‐34a targeted DKK1 to influence the activation of Wnt/β‐catenin signalling, thereby regulating cardiomyocyte hypertrophy.

### 3.5 TUG1 overexpression attenuated cardiac hypertrophy in vivo

**Figure 5:**
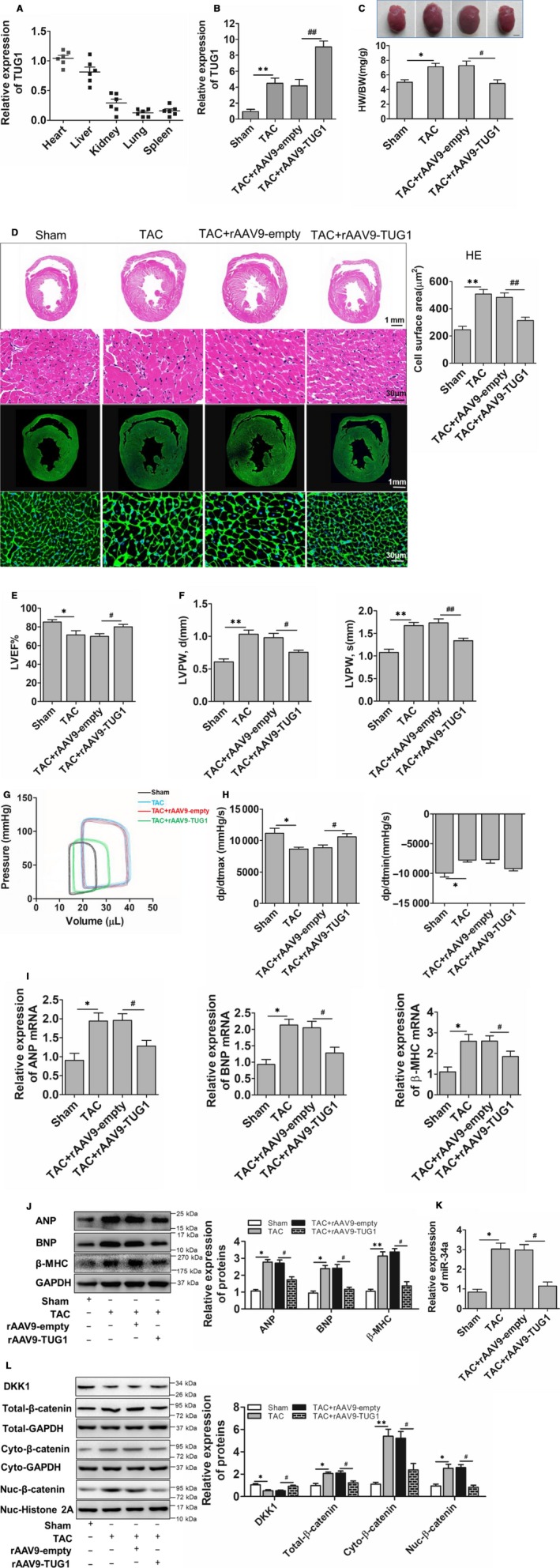
TUG1 alleviated cardiac hypertrophy in vivo. A, Real‐time PCR analyses for TUG1 expression in the tissues of normal mice mediated by rAAV9. (n = 6). B, Real‐time PCR analyses for TUG1 expression in the heart of mice treated by TAC surgery or/and rAAV9‐TUG1. (n = 6, **P < .01 vs Sham group, ##*P* < .01 vs TAC + rAAV9‐empty group). C, The ratio of heart weight to body weight (HW/BW) in the mice treated by TAC or/and rAAV9‐TUG1. (n = 6, bar = 2.0 mm). D, H&E and WGA staining for the heart of mice treated by TAC or/and rAAV9‐TUG1 and the quantified cell area results. (bar = 100 μm, **P < .01 vs Sham group, ##*P* < .01 vs TAC + rAAV9‐empty group). E,F, Echocardiography analysis of cardiac function in the mice treated by TAC or/and rAAV9‐TUG1. (n = 6, *P < .05 vs Sham group, **P < .01 vs Sham group, #*P* < .05 vs TAC + rAAV9‐empty group, ##*P* < .01 vs TAC + rAAV9‐empty group, LVEF, left ventricular ejection fraction; LVPW, d: left ventricle posterior wall thickness at diastole; LVPW, s: left ventricle posterior thickness at systole). G, Pressure‐volume loops measured with the Millar cardiac catheter system in the mice treated by TAC or/and rAAV9‐TUG1. H, Haemodynamic parameters in the mice treated by TAC or/and rAAV9‐TUG1. (n = 6, *P < .05 vs Sham group, #*P* < .05 vs TAC + rAAV9‐empty group; dp/dtmax: peak instantaneous rate of left ventricular pressure increase; dp/dtmin: peak instantaneous rate of left ventricular pressure decline). I, Real‐time PCR analyses for the mRNA expressions of hypertrophic biomarkers in the heart of mice treated by TAC or/and rAAV9‐TUG1. (n = 6, *P < .05 vs Sham group, #*P* < .05 vs TAC + rAAV9‐empty group). J, Western blot examination for the protein expressions of hypertrophic biomarkers in the heart of mice treated by TAC or/and rAAV9‐TUG1. (n = 6, *P < .05 vs Sham group, **P < .01 vs Sham group, #*P* < .05 vs TAC + rAAV9‐empty group). K, Real‐time PCR analyses for miR‐34a expression in the heart of mice treated by TAC or/and rAAV9‐TUG1. (n = 6, *P < .05 vs Sham group, #*P* < .05 vs TAC + rAAV9‐empty group). L, Western blot examination for DKK1 expression and the activation of the Wnt/β‐catenin signalling in the hearts of mice treated by TAC or/and rAAV9‐TUG1. (n = 6, *P < .05 vs Sham group, **P < .05 vs Sham group, #*P* < .05 vs TAC + rAAV9‐empty group)

To confirm the protective role of TUG1 on cardiac hypertrophy in vivo, the type 9 recombinant adeno‐associated virus (rAAV9) vector was employed to overexpress TUG1. GFP, serving as a reporter gene maker, was constructed into the rAAV9‐TUG1 to verify the infection efficiency of rAVV9 in hearts. As shown in Figure S3A, compared with control group, almost 96% cells in the heart were infected by rAVV9. Besides, rAAV9 induced TUG1 expression mainly in the hearts and livers of mice (Figure 5A). The up‐regulation of TUG1 induced by TAC was further enhanced by rAAV9‐TUG1 treatment (Figure 5B). Further, rAAV9‐TUG1 had no effect on heart weight (Figure S3B), cardiac function (Figure S3C‐E) and structure (Figure S3F) of the normal mice, but alleviated cardiac hypertrophy as anticipated, manifested by the decreased ratio of heart weight to body weight and heart size (Figure 5C), reduced cross‐sectional area and cell surface area (Figure 5D), improved LVEF% and decreased diastolic left ventricular posterior thickness (LVPW, d and LVPW, s) (Figure 5E,F and Table S4). Steady‐state pressure‐volume loops revealed worse heart function of TAC‐treated mice when compared with sham group‐with a rightwards and upwards shift of the PV loop. TUG1 overexpression resulted in a leftwards and downwards shift of the PV loop, indicating an improvement in systolic function (Figure 5G). Consistently, forced expression of TUG1 ameliorated cardiac dysfunction by increasing dp/dtmax and decreasing dp/dtmin (Figure 5H and Table S5). The up‐regulated mRNA and protein expressions of hypertrophic biomarkers were also inhibited by rAAV9‐TUG1 (Figure 5I,J). Next, the downstream pathway of TUG1 was examined in vivo. As shown in Figure 5K, TUG1 overexpression suppressed miR‐34a up‐regulation induced by TAC surgery. TUG1 overexpression also blocked the down‐regulation of DKK1 and activation of Wnt/β‐catenin signalling induced by TAC (Figure 5L). All of these results indicated that TUG1 overexpression alleviated TAC‐induced cardiac hypertrophy and dysfunction through TUG1/miR‐34a/DKK1/Wnt‐β‐catenin signalling in vivo.

## 4 DISCUSSIONS

**Figure 6:**
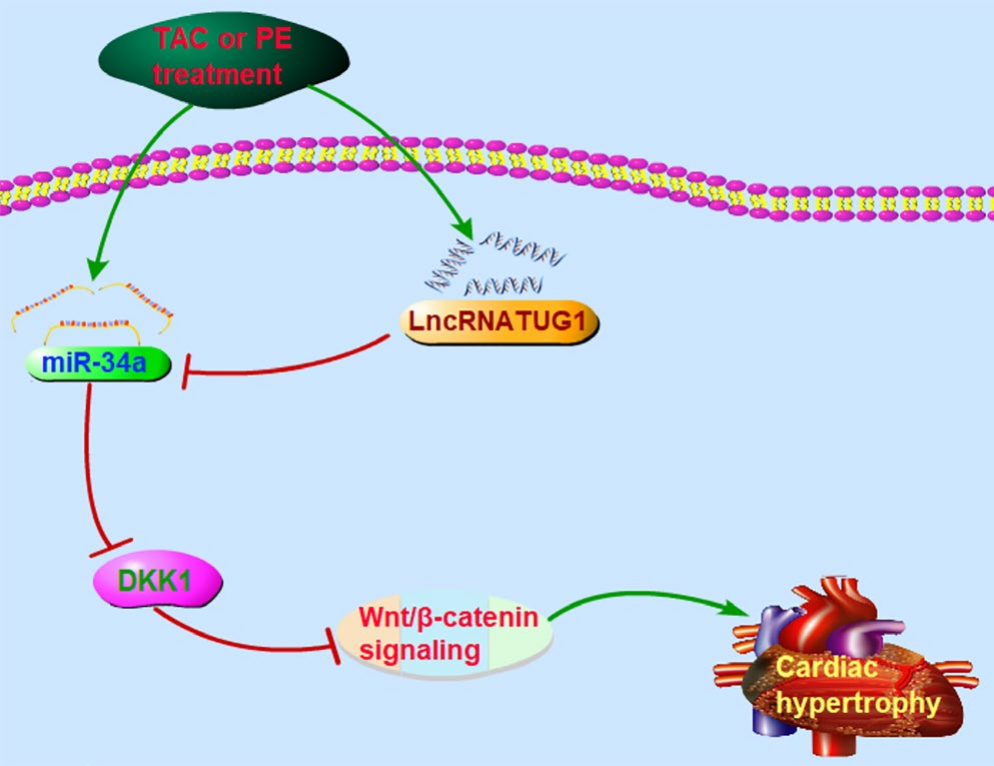
Schematic diagram shows the proposed signalling pathway linking lncRNA TUG1 to cardiac hypertrophy. Hypertrophic stimulation induces the up‐regulation of TUG1 and miR‐34a. Up‐regulation of miR‐34a targets DKK1 to block the inhibitory effects of DKK1 on Wnt/β‐catenin signalling, inducing cardiac hypertrophy. Up‐regulation of TUG1 enhances its ceRNA action on miR‐34a to alleviate the target regulation of miR‐34a on DKK1 and increase the expression of DKK1, thereby suppressing the activation of Wnt/β‐catenin signalling and relieving cardiac hypertrophy

In this study, we demonstrated TAC surgery and PE‐induced up‐regulation of TUG1 and miR‐34a. Up‐regulation of miR‐34a targeted DKK1 to block the inhibitory effects of DKK1 on Wnt/β‐catenin signalling, inducing cardiac hypertrophy. Up‐regulation of TUG1 caused by hypertrophic stimulation enhanced its ceRNA action on miR‐34a to alleviate the target regulation of miR‐34a on DKK1 and increase the expression of DKK1, suppressing the activation of Wnt/β‐catenin signalling and relieving cardiac hypertrophy (a schematic diagram shown in Figure 6).

LncRNAs serve as a vital factor in regulating pathological cardiac hypertrophy. For instance, lncRNA ROR (retinoic acid receptor‐related orphan receptor) interacts with miR‐133 to facilitate cardiac hypertrophy.^27^ LncRNA CHRF (cardiac hypertrophy related factor) induces cardiac hypertrophy through competitively binding miR‐93 to increase AKT3 expression.^28^ LncRNA CASC15 (cancer susceptibility candidate 15) promotes cardiac hypertrophy through sponging miR‐432‐5p to up‐regulate TLR4 expression.^29^ We demonstrated that lncRNA TUG1 ameliorated cardiac hypertrophy by sponging miR‐34a. Interestingly, we found that the up‐regulation of a lncRNA can also exert a protective role. We proposed that, in the initial period of cardiac hypertrophy, TUG1 up‐regulation may be an adaptive response to partially offset the increase of miR‐34a which promoted cardiac hypertrophy, leading to adaptively hypertrophic growth of cardiomyocytes. With sustaining hypertrophic stimulation, the up‐regulation of miR‐34a exceeded a certain threshold value. The increased expression of TUG1 was not enough to counteract the pro‐hypertrophy effect of miR‐34a, therefore causing cardiac hypertrophy. We, therefore, treated mice by rAAV9‐TUG1 through tail intravenous injection to overexpress TUG1. We found a high expression of TUG1 at the point of 2 weeks after TAC treatment could attenuate hypertrophic growth. However, 2‐week post‐TAC is an early time point. Therefore, in this study, we just focused on the early molecular mechanisms of TUG1, while the role of TUG1 overexpression in the later period of cardiac hypertrophy cannot be concluded. Also, the current study revealed that the up‐regulation of TUG1 prevented the hearts against pathological hypertrophy to a certain extent. In this study, the rAAV9 vector delivers TUG1 mainly in the hearts. However, rAAV9‐delivered TUG1 could also enrich in other organs, such as livers. Therefore, the possible systemic effects of TUG1 should not be excluded. Next, we will build heart‐specific mice to overexpress or knockout TUG1 expression, further confirming the role of TUG1 on cardiac hypertrophy.

Besides regulating cardiac hypertrophy which we have just reported, TUG1 is also reported to mediate cardiac injury. In ischaemia/hypoxia‐treated cardiomyocytes, TUG1 stimulates autophagic cell apoptosis by targeting miR‐142‐3p and up‐regulating HMGB1 and Rac1.^30^ Knockdown of TUG1 protects oxygen‐glucose deprivation followed by reperfusion (OGD/R)‐induced myocardial ischaemia‐reperfusion injury by inhibiting HMGB1 expression.^31^ TUG1 promotes the apoptosis and reduced the viability, migration and invasion of hypoxia‐treated cardiomyocytes.^32^ Interestingly, in H9c2 cardiomyocytes stressed with inflammation stimuli, TUG1 inhibits apoptosis and inflammatory response,^33^ while in the same cells under hypoxic conditions, TUG1 promotes apoptosis and aggravates a hypoxia‐induced injury,^32^ suggesting that the dichotomous effects of TUG1 are dependent on cell types as well as stimulating factors.

MicroRNA‐34 family members (miR‐34a, ‐34b and ‐34c) were reported to be up‐regulated in the heart in response to stress, and inhibition of these members provides therapeutic benefit in mice with pre‐existing pathological cardiac remodelling and dysfunction due to myocardial infarction (MI) or pressure overload.^34^ MiR‐34a, a member of this family, is reported to be a significant mediator in cardiac remodelling. Inhibition of miR‐34a attenuates cardiac dysfunction in a setting of moderate, but not severe, hypertrophic cardiomyopathy.^22^ In Angiotensin II‐induced cardiac hypertrophy, miR‐34a inhibits ATG9A expression and autophagy activity.^35^ During heart failure, miR‐34a inhibition alleviates RBFox2 depletion‐induced heart dysfunction.^36^ Besides, miR‐34a regulates cardiomyocyte apoptosis. MiR‐34a contributes to high glucose‐induced decreases in Bcl‐2 expression and subsequent cardiomyocyte apoptosis. MiR‐34a aggravates coxsackievirus B3‐induced cardiomyocyte apoptosis through the SIRT1‐p53 pathway.^37^ In our study, we reported miR‐34a was up‐regulated in PE‐treated cardiomyocytes and in hypertrophic hearts of mice induced by TAC; TUG1 sponged and sequestered miR‐34a to attenuate the inhibitory effect of miR‐34a on DKK1, which prevented cardiac hypertrophy. It is indicated miR‐34a is a significant modulator and plays a different regulating role in different pathological processes of heart diseases.

Wnt/β‐catenin signalling has been reported to be an essential pathway in mediating cardiac hypertrophy.^24^ In Angiotensin II‐treated cardiomyocytes, multiple Wnt ligands were induced to activate β‐catenin and stimulate the expressions of hypertrophic biomarkers, thereby leading to hypertrophy response.^24^ We reported that the Wnt/β‐catenin signalling was activated in cardiac hypertrophy. Indeed, to the best of our knowledge, this was the first time to report DKK1 exerted an inhibitory effect of the Wnt/β‐catenin signalling pathway in PE‐ and TAC surgery‐induced cardiac hypertrophy.

In conclusion, our research revealed TUG1 targeted miR‐34a to block the inhibitory effects of miR‐34a on DKK1, thereby inhibiting the activation of Wnt/β‐catenin signalling and alleviating cardiac hypertrophy.

## CONFLICT OF INTERESTS

The authors declare that they have no competing or financial interests.

## AUTHOR CONTRIBUTIONS

Xiaozhou Zou, Qingxia Fang, Ting Liu and Chenhuan Yu performed the study, analysed, interpreted the data and wrote the manuscript. Xiaozhou Zou, Xiuli Yang, Yanfei Shao, Jiana Shi and Xiaolan Ye. contributed to acquisition of data and manuscript preparation and revision. Xiaozhou Zou, Xiaochun Zheng, Jieping Yan and Danfeng Xu conceived the hypothesis, participated in the experimental design, data interpretation and manuscript preparation and revision. All authors approved the final version of the manuscript.

## Data Availability

The data that support the findings of this study are available from the corresponding author upon reasonable request.
