# Supplementary material for: LncRNA TUG1 alleviates cardiac hypertrophy by targeting miR‐34a/DKK1/Wnt‐β‐catenin signalling
Source: J Cell Mol Med. 2020 Feb 14;24(6):3678–91. doi: 10.1111/jcmm.15067 (PMC7131932; doi:10.1111/jcmm.15067)
Supplement: Supplementary file 1 [file JCMM-24-3678-s001.doc]

**LncRNA TUG1 alleviates cardiac hypertrophy by targeting miR-34a/DKK1/Wnt-β-catenin signaling**

**Qingxia Fang ^1,2 #^, Ting Liu ^3 #^, Chenhuan Yu ^4^, Xiuli Yang ^1,2^, Yanfei Shao ^1,2^, Jiana Shi ^1,2^, Xiaolan Ye ^1,2^, Xiaochun Zheng ^1,2^, Jieping Yan ^1,2^, Danfeng Xu ^1,2^, Xiaozhou Zou ^1,2 *^**

**Supporting information**

**1 Expanded Material and Methods (Online Data Supplement)**

***Animal Experiments***

C57BL/6 mice (male, 8 weeks old) were sourced from the Key Laboratory of Experimental Animal and Safety Evaluation, Zhejiang Academy of Medical Sciences (Zhejiang, China). Mice underwent a TAC surgery and were fed for 1 week, 2 week, 4 week and 8 week under standard animal room conditions to induce the cardiac pressure overload models as described in a previous study (12). Moreover, mice were injected with rAAV9 (4×10^11^ vector genomes (vg) / mouse) carrying an empty vector or TUG1 via tail vein. rAAV9-TUG1 was produced by Hanbio Biotechnology and stored at -80°C. Real-time quantitative assay was used to quantify TUG1 copy number. DNeasy Tissue Kits (Qiagen) was used to extract genomic AAV9-TUG1 vector DNA from the frozen cardiac tissues. To examine vector genome copies, 100 ng of each sample was applied in duplicate. The copy number standard range of plasmid DNA was 10-10^8^ copies/µl. 150 copies/µl DNA was the lower limit detection. Three weeks after injection, qPCR was used to monitor the levels of TUG1 in live, heart, kidney, lung and spleen tissues of mice carrying a vector or TUG1. The other mice carrying a vector or TUG1 underwent a TAC surgery and were fed for 2 weeks. Avertin (160 mg/kg) was injected intraperitoneally to anesthetize mice in the whole experiment. Hearts and other organs were collected and stored in liquid nitrogen. All the experimental procedures were performed in accordance to and approved by the Animal Ethics Committee of Zhejiang Provincial People's Hospital.

***Cell isolation and culture***

Cardiomyocytes were isolated from neonatal mice (1-2 days) and adult mice (male, 8 weeks old). In brief, after wash, hearts were cut up in saline with HEPES-buffer. Then, the tissues were decentralized and incubated in HEPES-buffered saline solution with 0.14 mg/ml collagenase and 1.2 mg/ml pancreatin at 37 ℃. The cells collected by centrifugation were re-suspended in Dulbecco’s modified Eagle medium/F-12 with 0.1 mM ascorbate, 5% heat-inactivated fetal bovine serum (FBS), 100 U/ml penicillin, insulin-transferring-sodium selenite media supplement, 0.1 mM bromodeoxyuridine and 100 μg/ml streptomycin. After pre-plated at 37︒C for 1 h, the cells were plated in dishes coated by 10 μg/ml laminin. For inducing hypertrophy, cells were treated with PE at 100 nM for 12h, 24h and 48h.

Cardiac fibroblasts were isolated from neonatal mice (1-2 days) and adult mice (male, 8 weeks old). After wash, hearts were mounted via the aorta onto a 27-gauge cannula attached to a Langendorff-type apparatus for retrograde perfusion of the coronary arteries. Subsequently, hearts were perfused with sterile calcium-free Krebs-Ringer bicarbonate buffer for 5 min and KRB enzyme solution containing 1 mg/ml fatty-acid free albumin, 2.5 mM CaCl2 and 0.5 mg/ml type II collagenase for 20 min, in turn. Next, after cut up in KRB enzyme solution containing 10 mg/ml albumin, the tissues were filtered and collected by centrifugation at 25 g for 5 min to remove debris, red blood cells and cardiomyocytes. Then, the supernatant was centrifuged at 1000g for 10 min, and the cells were harvested, re-suspended and plated into dishes with RPMI 1640 medium containing 10% heat-inactivated FBS and antibiotics. Finally, 4 h later, non-adherent cells were removed by aspiration.

***Cell transfection***

cDNA encoding TUG1 and DDK1 were synthesized and sub-cloned into the XhoI and BamHI sites of the pLVX-IRES-Neo plasmid vector to construct the pLVX-TUG1 and pLVX-DKK1 vector. This process was provided and carried out by Gene Chem (Shanghai, China). Before viral infection, cells were plated in a 24-well cell culture plate at the number of 0.5 × 10^5^ cells per well. After culturing for 24 h, the cells were infected by adding the viral stock at a multiplicity of infection (MOI) of 100. The culture condition is 37 °C and 5% CO_2_; after 24h, in order to avoiding cell toxicity, normal complete medium was used to replace the transfection mixture. After incubating for 48h, these cells were added by PE for 24h. TUG1 siRNA, miR-34a mimic, miR-34a inhibitor, DKK1 siRNA, and the negative control were manufactured by RIBOBIO (Guangzhou, China). TUG1 siRNA (si-TUG-1, si-TUG-2, si-TUG-3, 100 nM), miR-34a mimic (75 nM, 150 nM, 300 nM), miR-34a inhibitor (50 nM, 100 nM, 200 nM), DKK1 siRNA (si-DKK1-1, si-DKK1-2, si-DKK1-3, 125nM) were respectively transfected into cardiomyocyte by lipofectamine 2000 (Invitrogen, Carlsbad, CA, USA) according to the manufacturer’s instructions prior to PE treatment for 24 h.

***Real-time PCR analysis***

Trizol reagent (Invitrogen, USA) was used to extract total RNA from cardiomyocytes and cardiac tissues. After total RNA extracting, the Prime Script reverse transcription reagent Kit (CWBIO, China) was used to produce cDNA. An ABI 7300 real-time PCR system was used to perform Real-time PCR process. The reagent was SYBR Green PCR Master Mix kit (TOYOBO, Japan). The primer sequences of this study were shown in Supplementary Table 1. U6 and GAPDH served as the internal reference genes. Comparative threshold cycle method was used to count the relative expression of real-time PCR products.

***Cell immunostaining analysis***

4% formaldehyde was used to fix cardiomyocytes for 1 h. Then 0.5% Triton-X 100 was used to treat the fixed cells for 15 min at room temperature. Next, cells were treated with α-actinin primary antibody (Supplementary Table 2) overnight at 4︒C. After elution, cells were treated with fluorescence-conjugated secondary antibody. Nikon laser microscope (Eclipse E600, Nikon Instruments Inc., Japan) was used to capture photos.

***Protein/DNA ratio detection***

After treatment, cardiomyocytes were washed with PBS. Then, the cells was incubated with perchloric acid and collected by centrifugation (10000×g, 10 min). After centrifugation, the precipitates were treated with KOH for 30 min at 70 ℃. Lowry method Hoechst dye 33258 was used to examine Protein and DNA contents.

***Western blot examination***

Lysis buffer (Beyotime Institute of Biotechnology, China) was used to extract total cellular protein. The Nuclear and Cytoplasmic Protein Extraction Kit (Beyotime) was used to detect β-catenin proteins in the cytoplasm as well as nucleus. The proteins were subjected to 6%, 10%, and 12% SDS-PAGE and transferred to polyvinylidene fluoride membrane. After blocking with 5% BSA, the membranes were incubated with primary antibodies (Supplementary Table 3) at 4︒C for overnight. Then the membranes were washed by TBST and incubated with HRP-conjugated goat anti-mouse or anti-rabbit IgG for 1 h at room temperature. According to the manufacturer's recommendations, enhanced chemiluminescence was used to visualize the target proteins. Density analysis was used to quantify the expression of target protein against GAPDH.

***Histological analysis***

4% paraformaldehyde was used to collect and fix the mice heart tissues. After 24 h, the fix hearts was embedded into paraffin according to standard histological protocols. Next, the tissues were cut into cross-sectional slices of 5 mm thick. Then, the slices were stained by HE or WGA staining to examine histopathology.

***Luciferase reporter assay***

PCR was used to amplify the sequence of TUG1 or DKK1 including the predicted miR-34a binding sequence. Next the sequence was inserted into a pmirGLO Dual-luciferase Target Expression Vector (Promega, Madison, WI, USA) to produce Wt-TUG1 or Wt-TUG1 reporter vector. In order to examine the specificity binding, the miR-34a seed region binding site was mutated to build the corresponding mutant, which was named as Mut-TUG1 or Mut-DKK1. Wt-TUG1/Mut-TUG1 or Wt-DKK1/Mut-DKK1 was co-transfected with miR-34a mimic, mimic Negative Control, miR-34a inhibitor or inhibitor Negative Control into cardiomyocytes using Lipofectamine 2000. Dual-Luciferase Reporter Assay System (Promega) was used to carry out Luciferase reporter assay. Renilla luciferase activity was used to measure and normalize the firefly luciferase activity.

**2 Supplementary Tables**

**Supplementary Table 1:** Mouse oligonucleotide primers for real-time PCR.

| Gene | Forward primer | | Reverse primer |
| --- | --- | --- | --- |
| U6 | | gcttcggcagcacatatactaa | aacgcttcacgaatttgcgt |
| TUG1 | | ggcacccagtgtaaagca | aagcagcagataacagagttga |
| miR-34a-5p | | acactccagctgggtggcagtgtcttagc | ctcaactggtgtcgtgga |
| GAPDH | | tgcccagaacatcatccct | ggtcctcagtgt agcccaag |
| ANP | | ctcccaggccatattggag | tccaggtggtctagcaggtt |
| BNP | | gctcttgaaggaccaaggcctcac | gatccgatccggtctatcttgtgc |
| β-MHC | | gtgaagggcatgaggaagagt | aggccttcaccttcagctgc |
| DKK1 | | acattcgccaccttctggattg | gcaaaagcaccaaccacacttg |

**Supplementary Table.2**: Characteristics of primary antibodies used in Immunohistochemistry studies.

| Target | Host specie | Supplier | Concentration |
| --- | --- | --- | --- |
| α-actinin | mouse | Sigma (Lot No. A7811) | 1:100 |

**Supplementary Table.3**: Characteristics of primary antibodies used in western blot studies.

| Target | Host specie | Supplier | Concentration |
| --- | --- | --- | --- |
| GAPDH | mouse | Beyotime Biotechnology | 1:2000 |
| ANP | mouse | Santa Cruz(sc-515701) | 1:1000 |
| BNP | Rabbit | Abcam (ab236101) | 1:500 |
| β-MHC | Rabbit | Abcam(ab207926) | 1:2000 |
| DKK1 | Rabbit | Abcam(ab61275) | 1:500 |
| β-catenin | Rabbit | Abcam (ab27798) | 1:1000 |
| Histone H2A | Goat | Abcam (ab140498) | 1:3000 |

**Supplementary Table 4:** Echocardiographic characteristics of Sham, TAC, TAC +

**rAAV9-empty, TAC+ rAAV9-TUG1**

|  | Sham  (n=7) | TAC  (n=7) | TAC+ rAAV9-empty  (n=7) | TAC+ rAAV9-TUG1  (n=7) |
| --- | --- | --- | --- | --- |
| HR (bpm) | 487 ± 36 | 517 ± 27 | 528 ± 31 | 519 ± 21 |
| LVEF (%) | 85.22± 5.61 | 71.40 ± 5.27^*^ | 69.81 ± 3.97^*^ | 80.17 ± 7.18^#^ |
| LVPW, d (mm) | 0.607 ± 0.101 | 1.031 ± 0.072^*^ | 0.979 ± 0.315^*^ | 0.755 ± 0.081^#^ |
| LVPW, s (mm) | 1.077 ± 0.257 | 1.674 ± 0.316^*^ | 1.732 ± 0.121^*^ | 1.337 ± 0.152^#^ |
| IVS, d (mm) | 0.603 ± 0.069 | 0.860 ± 0.117^*^ | 0.904± 0.097^*^ | 0.699 ± 0.059^#^ |
| IVS, s (mm) | 0.886 ± 0.092 | 1.166 ± 0.109^*^ | 1.229 ± 0.021^*^ | 0.938 ± 0.076^#^ |
| LVID, d (mm) | 2.998 ± 0.271 | 3.502 ± 0.082^*^ | 3.394 ± 0.194^*^ | 3.111 ± 0.105^#^ |
| LVID, s (mm) | 1.428 ± 0.175 | 2.198 ± 0.406^*^ | 2.074 ± 0.173^*^ | 1.775 ± 0.197^#^ |
| FS (%) | 45.89 ± 6.98 | 34.24 ± 4.17^*^ | 32.34 ± 6.89^*^ | 39.88 ± 5.02^#^ |

HR, heart rate; bpm, beat per minute; LVEF, left ventricular ejection fraction; LVPW, d, LV posterior wall thickness at diastole; LVPW, s, LV posterior wall thickness at systole; IVS, d, LV anterior wall thickness at diastole; IVS, s, LV anterior wall thickness at systole; LVID, d, LV internal diameter at diastole; LVID, s, LV internal diameter at systole; FS, fractional shortening. n=7, **P* < 0.05 *vs* Sham group, #P < 0.05 *vs* TAC + rAAV9-empty group

**Supplementary Table 5**: Comparison of hemodynamic variables for Sham, TAC, TAC+ rAAV9-empty, TAC+ rAAV9- TUG1.

|  | Sham  (n=7) | TAC  (n=7) | TAC+rAAV9-empty  (n=7) | TAC+ rAAV9-TUG1  (n=7) |
| --- | --- | --- | --- | --- |
| HR (bpm) | 499 ± 23 | 528 ± 18 | 509 ± 20 | 510 ± 29 |
| dp/dtmax (mmHg/s) | 11151 ± 271 | 8662±301^*^ | 8880 ± 170^*^ | 10578 ± 348^#^ |
| dp/dtmin (mmHg/s) | -9931 ± 426 | -7748 ± 389^*^ | -7663 ± 468^*^ | -9146 ± 219 |
| Pmax (mmHg) | 53 ± 14 | 41 ± 9^*^ | 40 ± 13^*^ | 49 ± 18^#^ |

HR, heart rate; bpm, beat per minute; dp/dtmax, peak instantaneous rate of left ventricular pressure increase; dp/dtmin, peak instantaneous rate of left ventricular pressure decline; Pmax, peak systolic pressure. n=7, **P* < 0.05 *vs* Sham group, #*P* < 0.05 *vs* TAC + rAAV9-empty group.

**3 Supplementary Figures (Online Data Supplement)**

**Fi****g.S1**


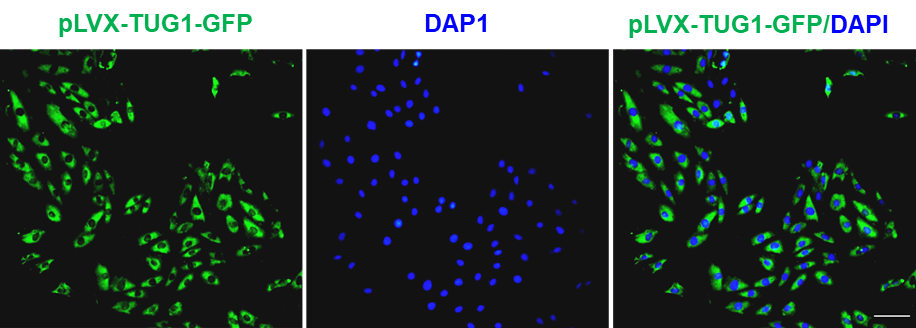
**A**


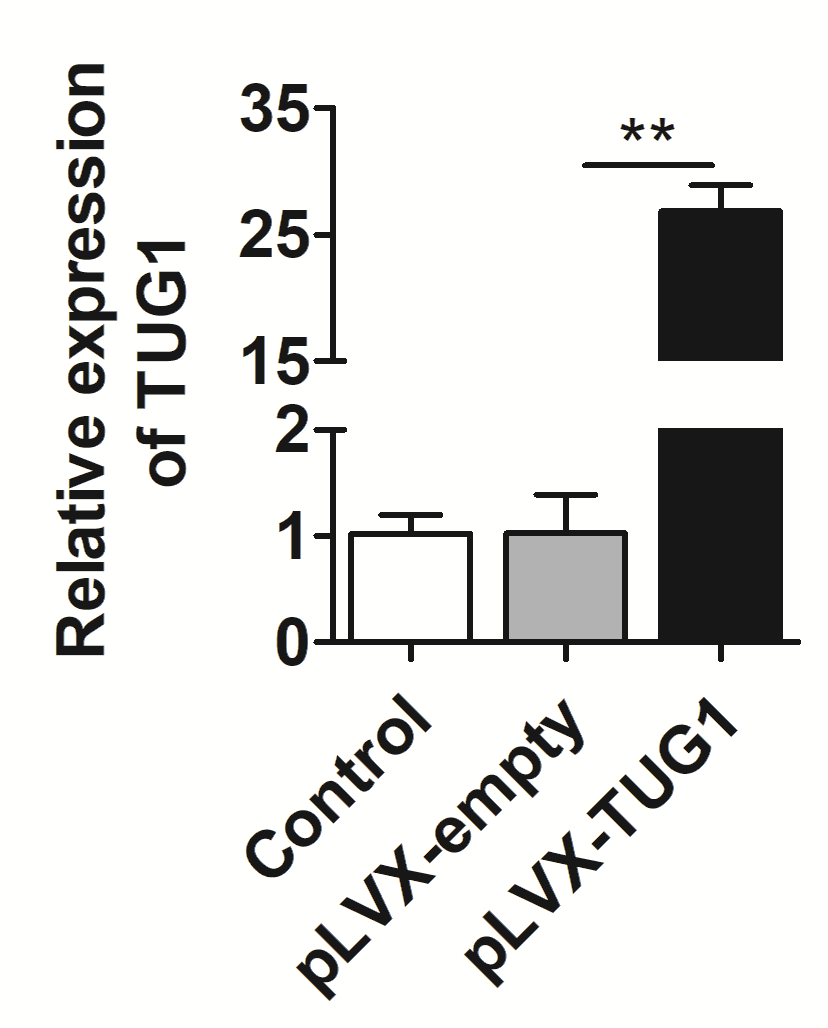

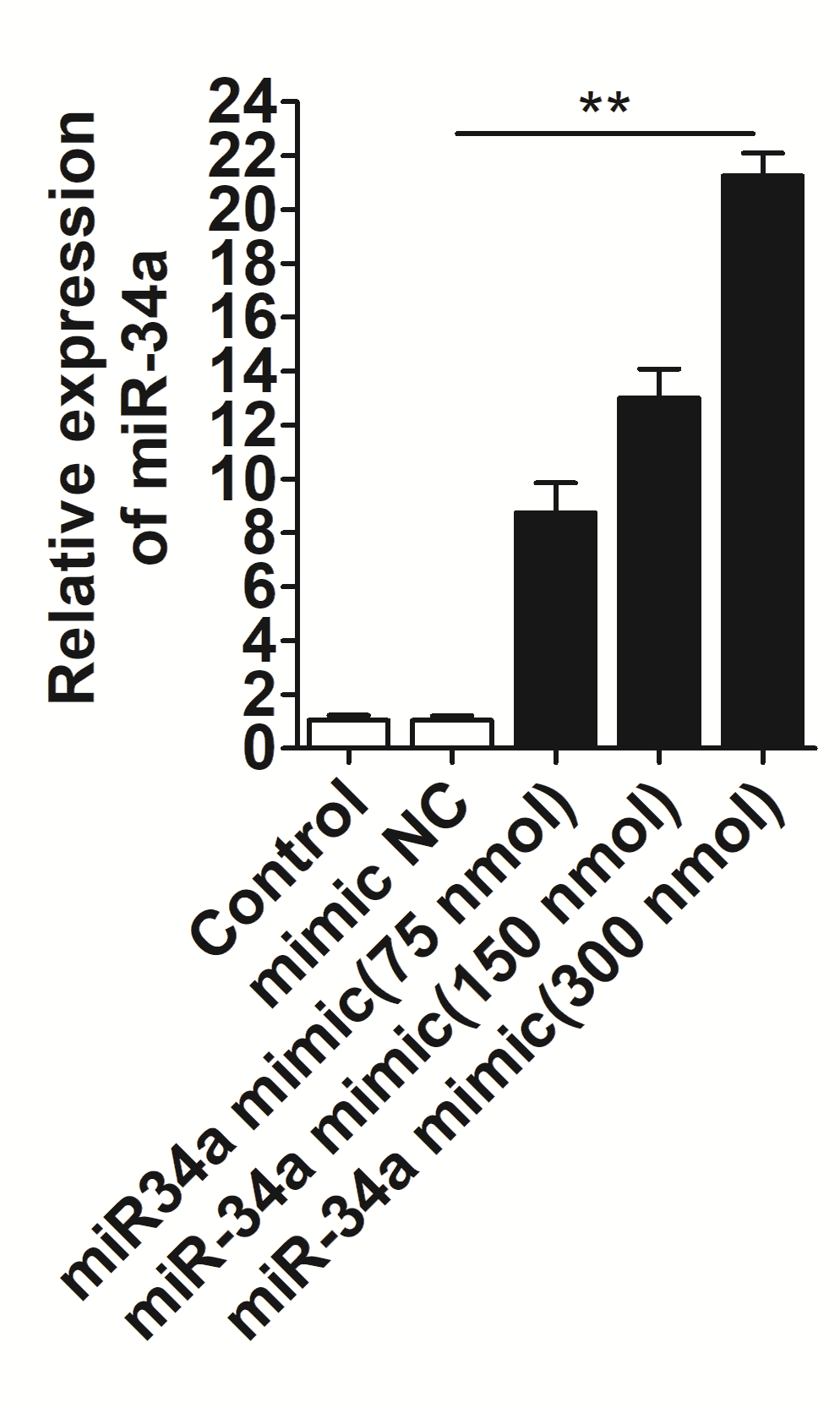

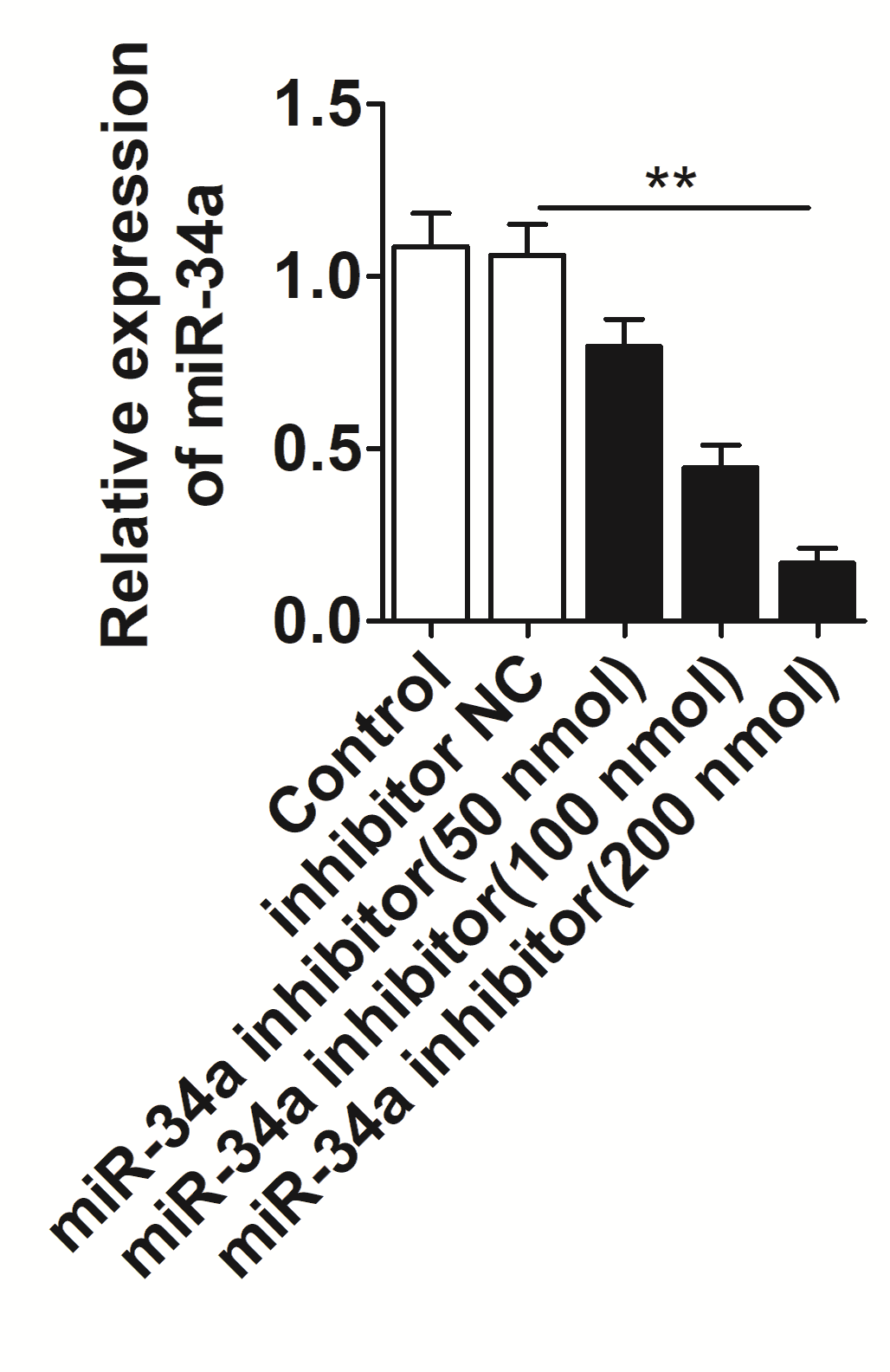
**B C D**


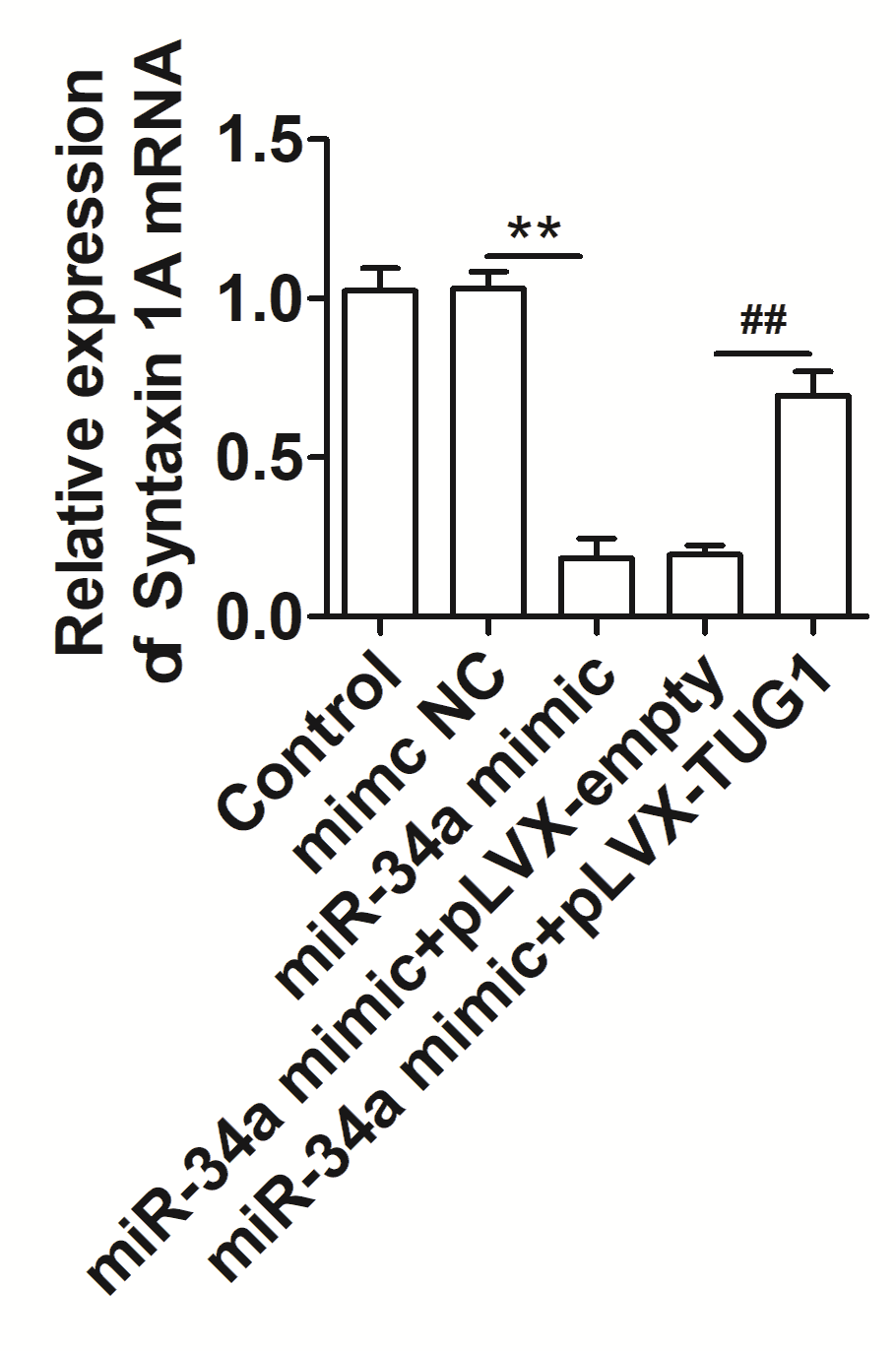

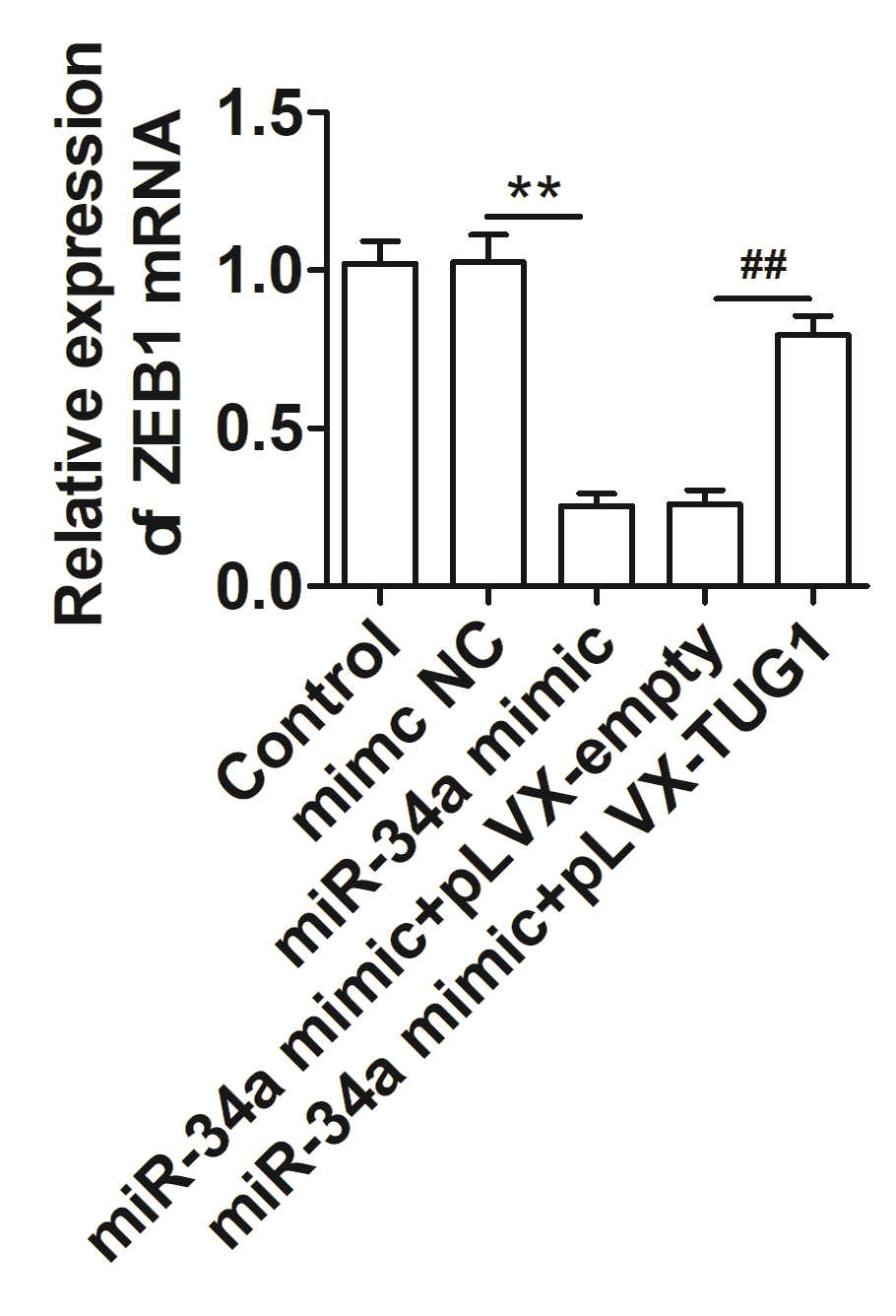

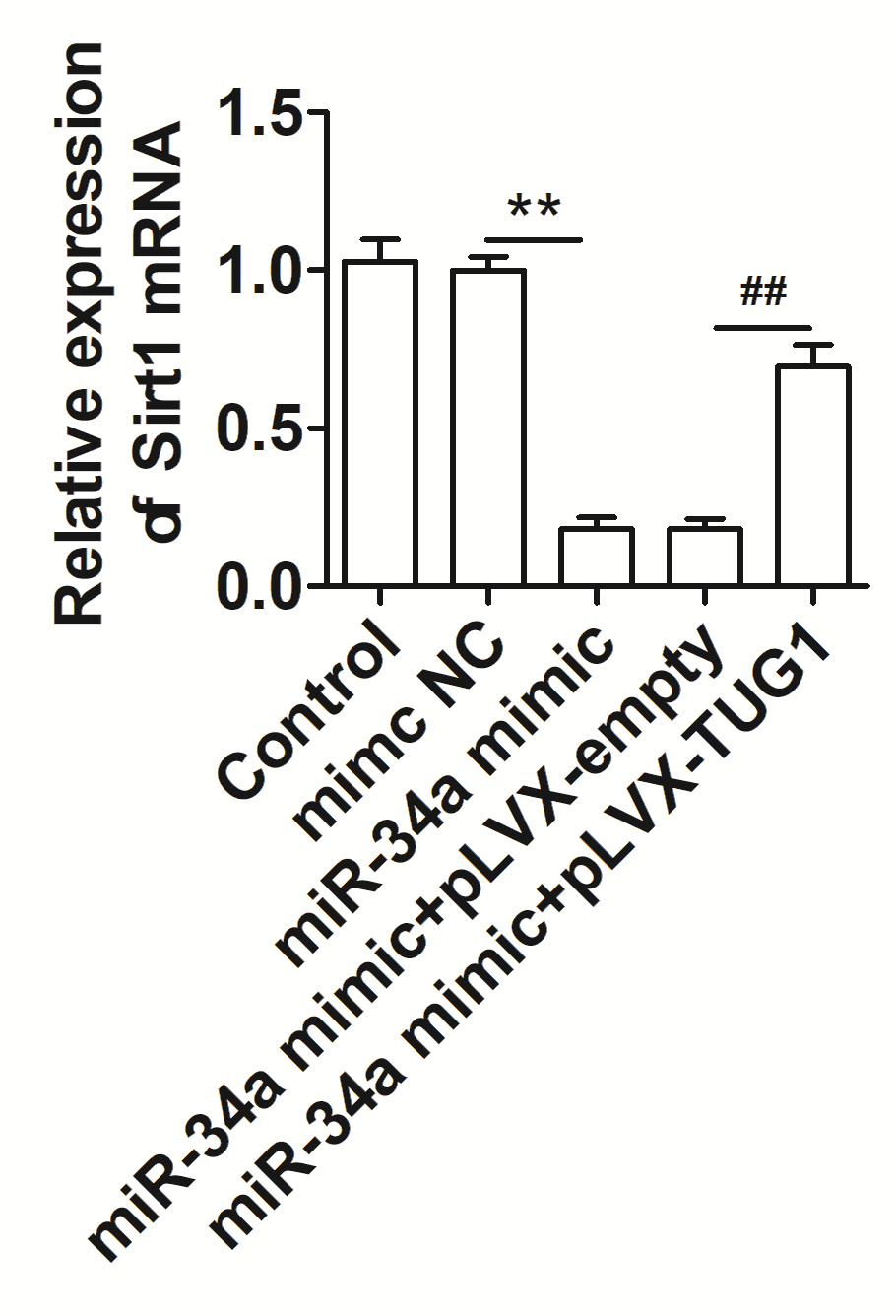
**E F G**

**Fig.S1**

**A:** Fluorescent microscopy examination for the transfection efficiency of pLVX-TUG1-GFP in cardiomyocytes, bar=50 μm. **B:** Real time-PCR analyses for overexpression efficiency of pLVX-TUG1 in cardiomyocytes. (n=3; ***P* < 0.01 *vs* pLVX-empty). **C:** Real time-PCR analyses for the interference efficiency of miR-34a inhibitor in cardiomyocytes. (n=3; ***P* < 0.01 *vs* inhibitor NC). **D:** Real time-PCR analyses for overexpression efficiency of miR-34a mimic in cardiomyocytes. (n=3; ***P* < 0.01 *vs* mimic NC). E-G: Real time-PCR analyses for mRNA expressions of other known miR-34a targets including Sirt1, ZEB1and Syntaxin 1A in cardiomyocytes treated by miR-34a mimic or/and pLVX-TUG1. (n=4; ***P* < 0.01 *vs* mimic NC, ## *P* < 0.01 *vs* mimic NC+pLVX-empty).

**Fig.S2**


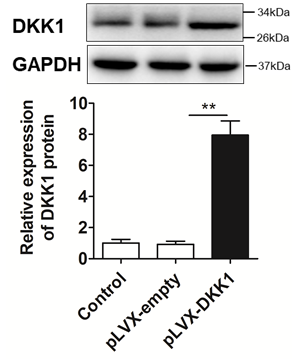

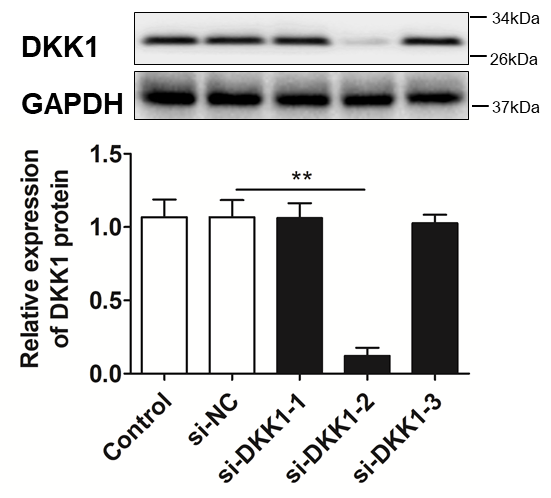
**A B**

**Fig.S2**

**A:** Western blot analyses for the interference efficiency of DKK1 siRNA in cardiomyocytes. (n=3; ***P* < 0.01 *vs* si-NC). **B:** Western blot analyses for overexpression efficiency of pLVX-DKK1 in cardiomyocytes. (n=3; ***P* < 0.01 *vs* pLVX-empty).

**Fig.S3**


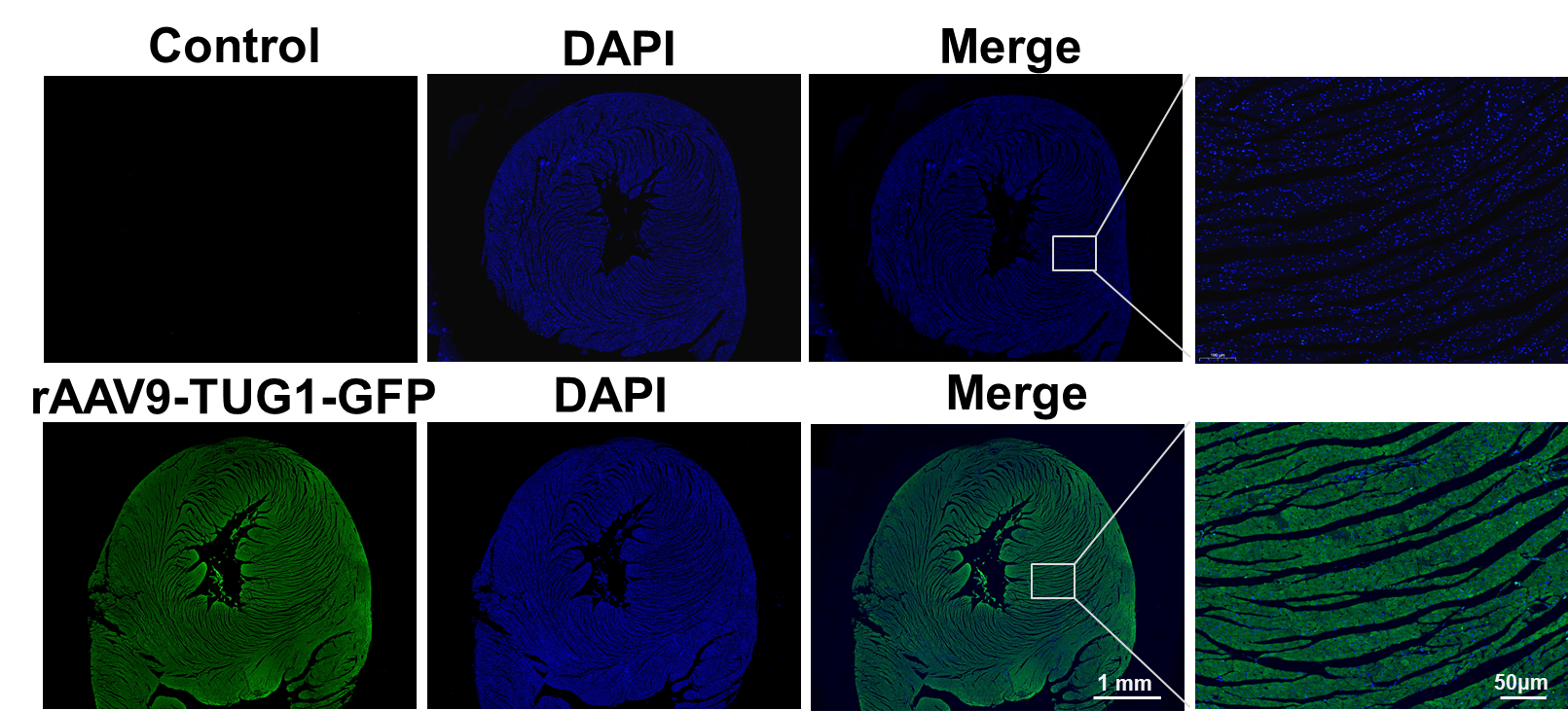
**A**


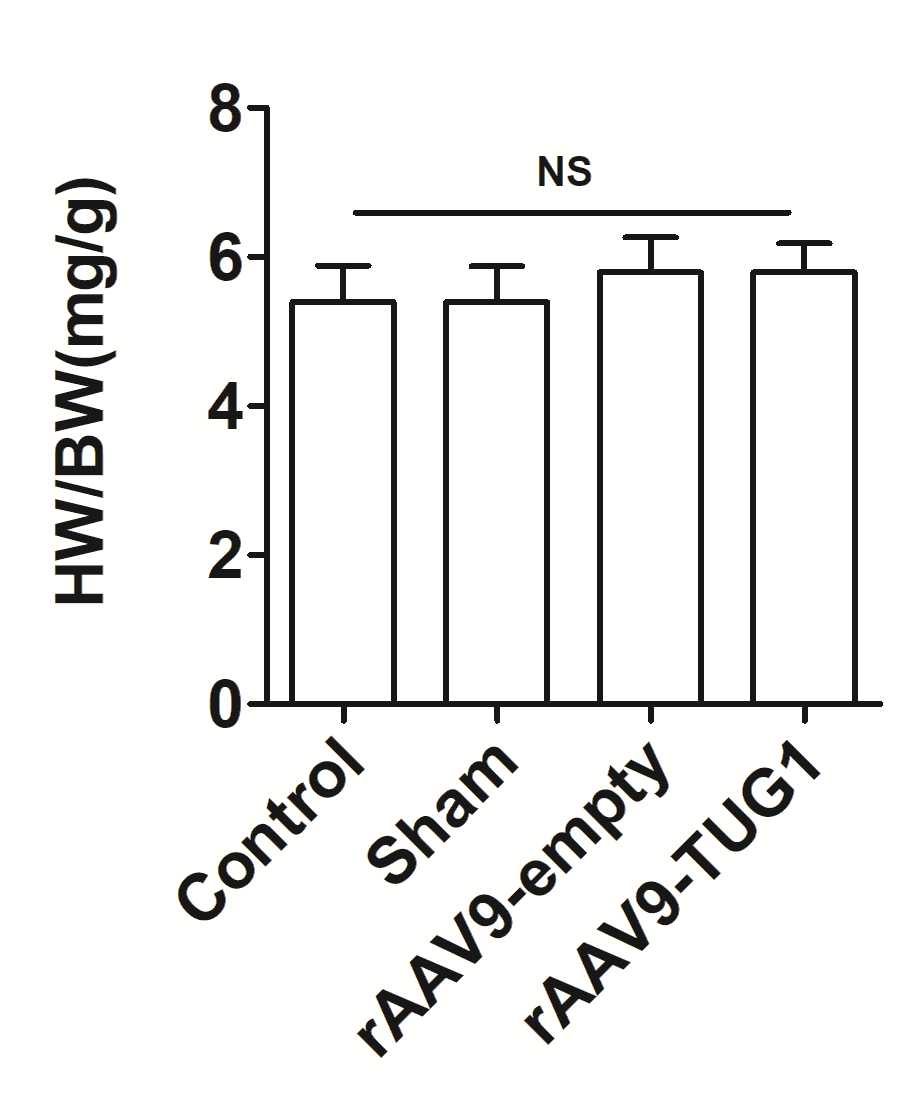

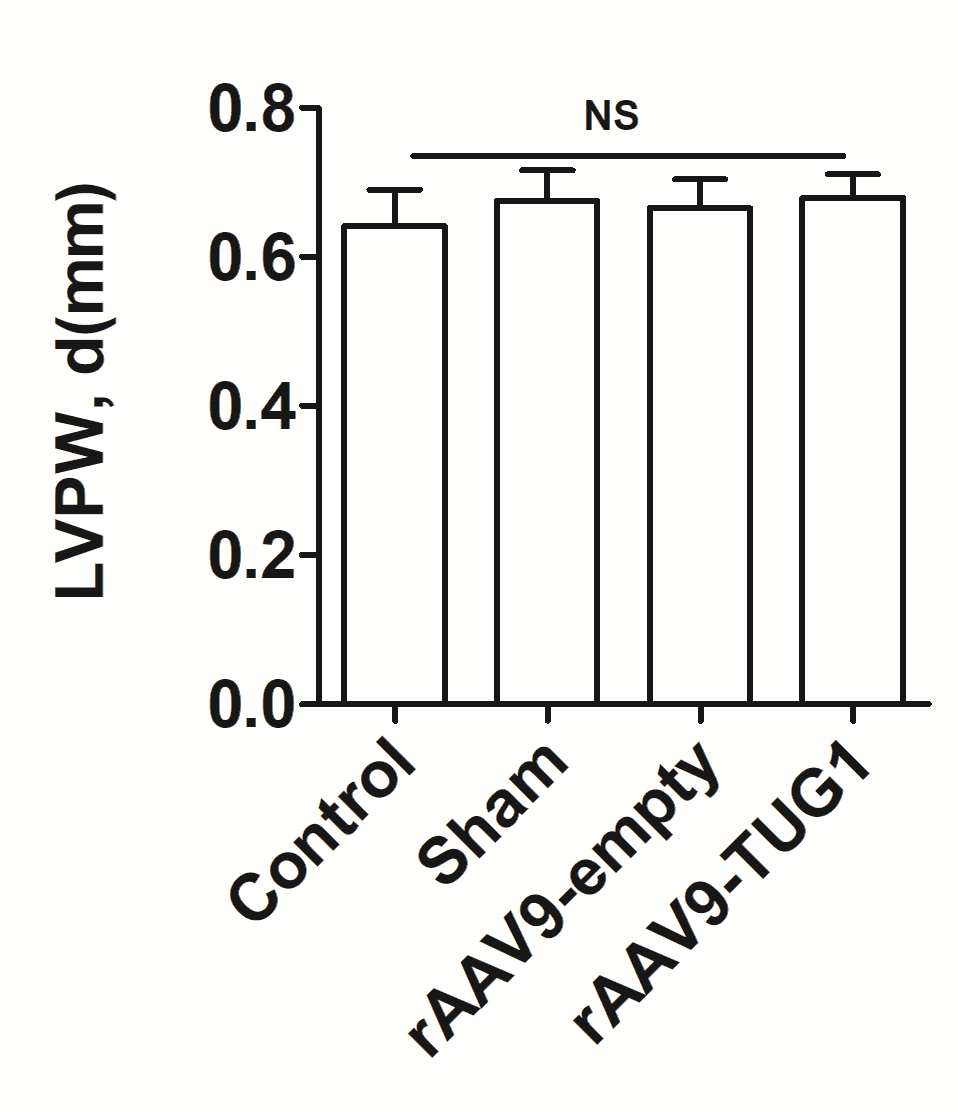

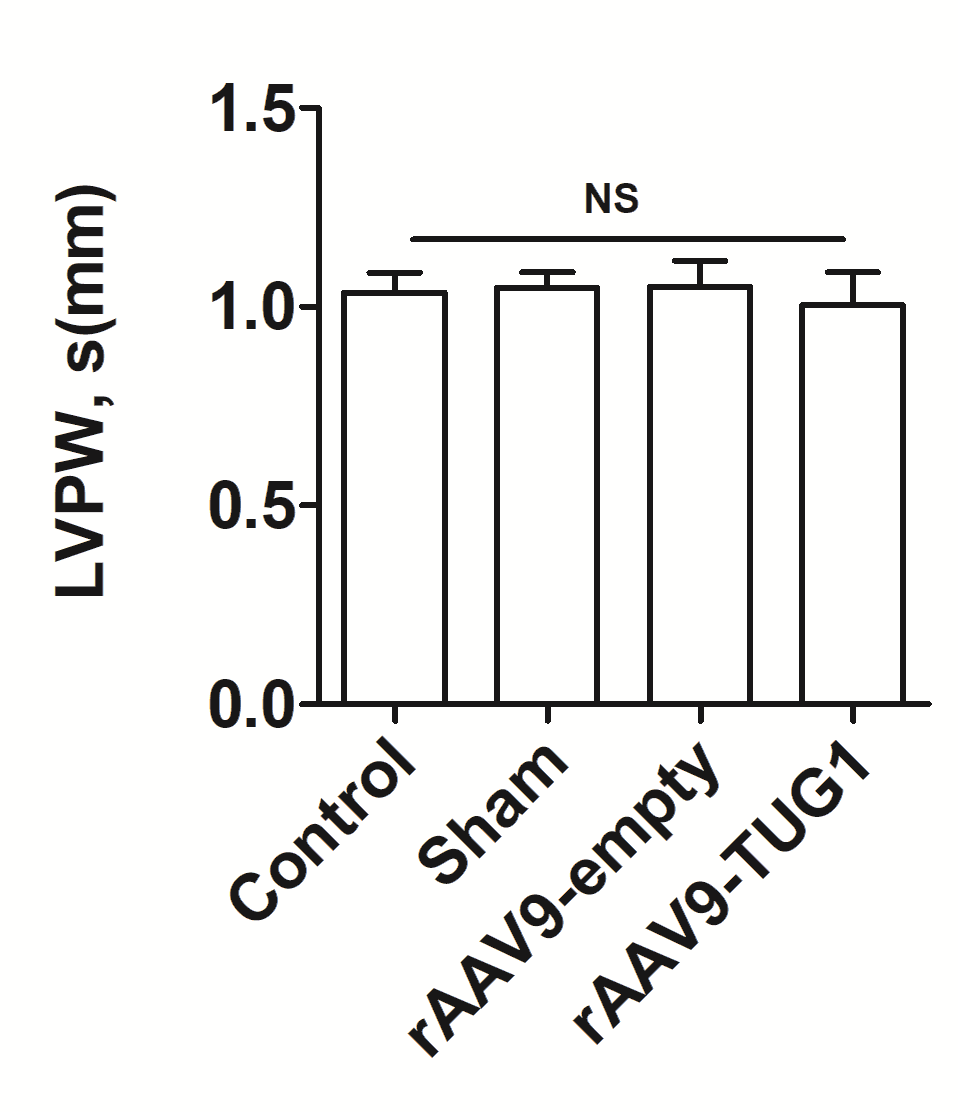
**B C**


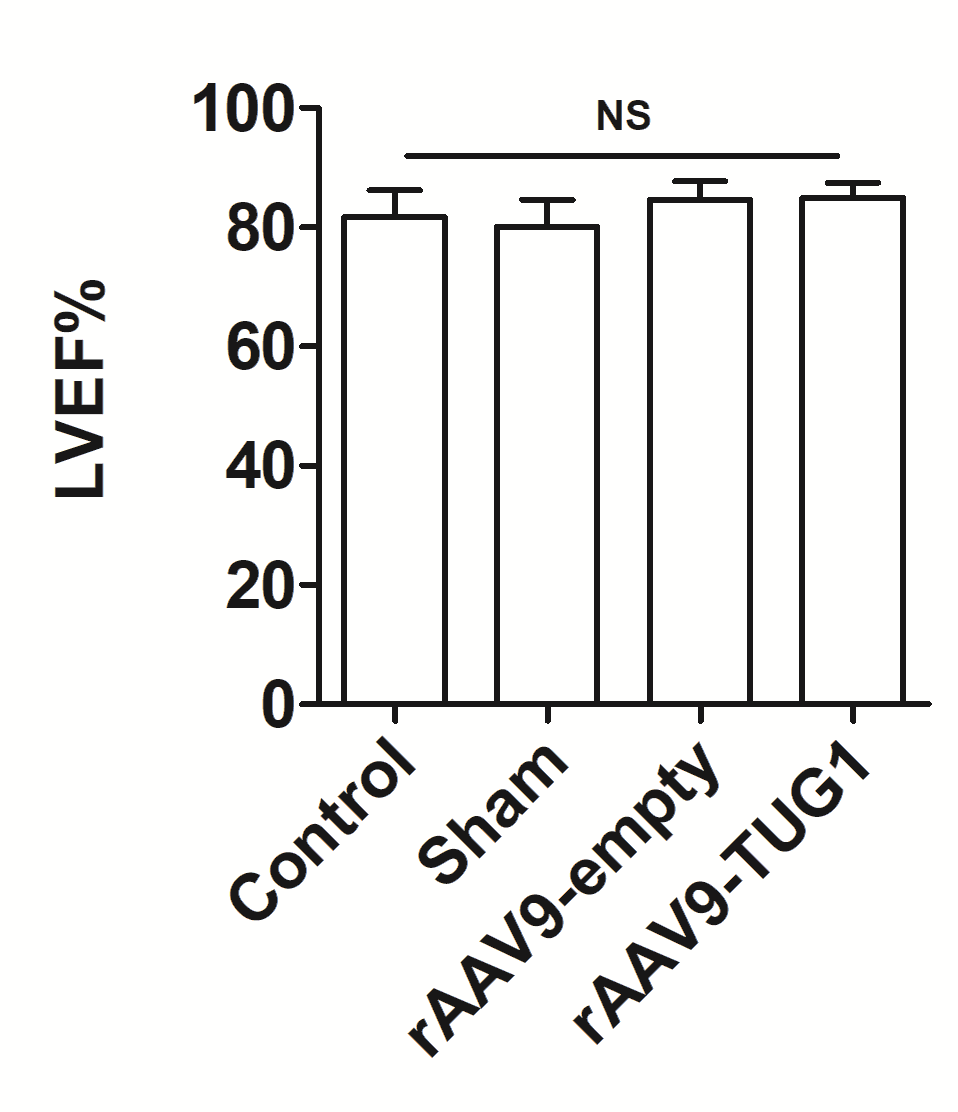

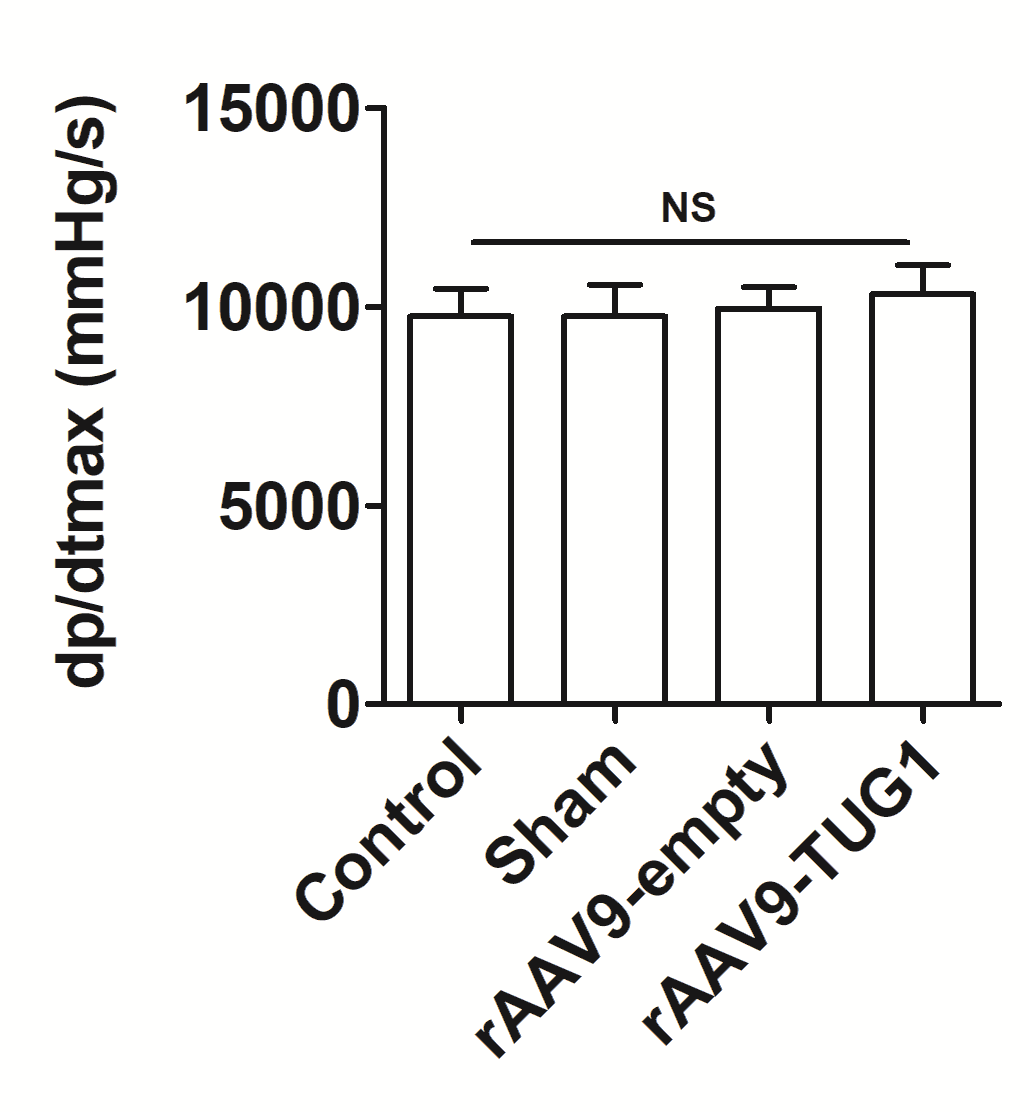

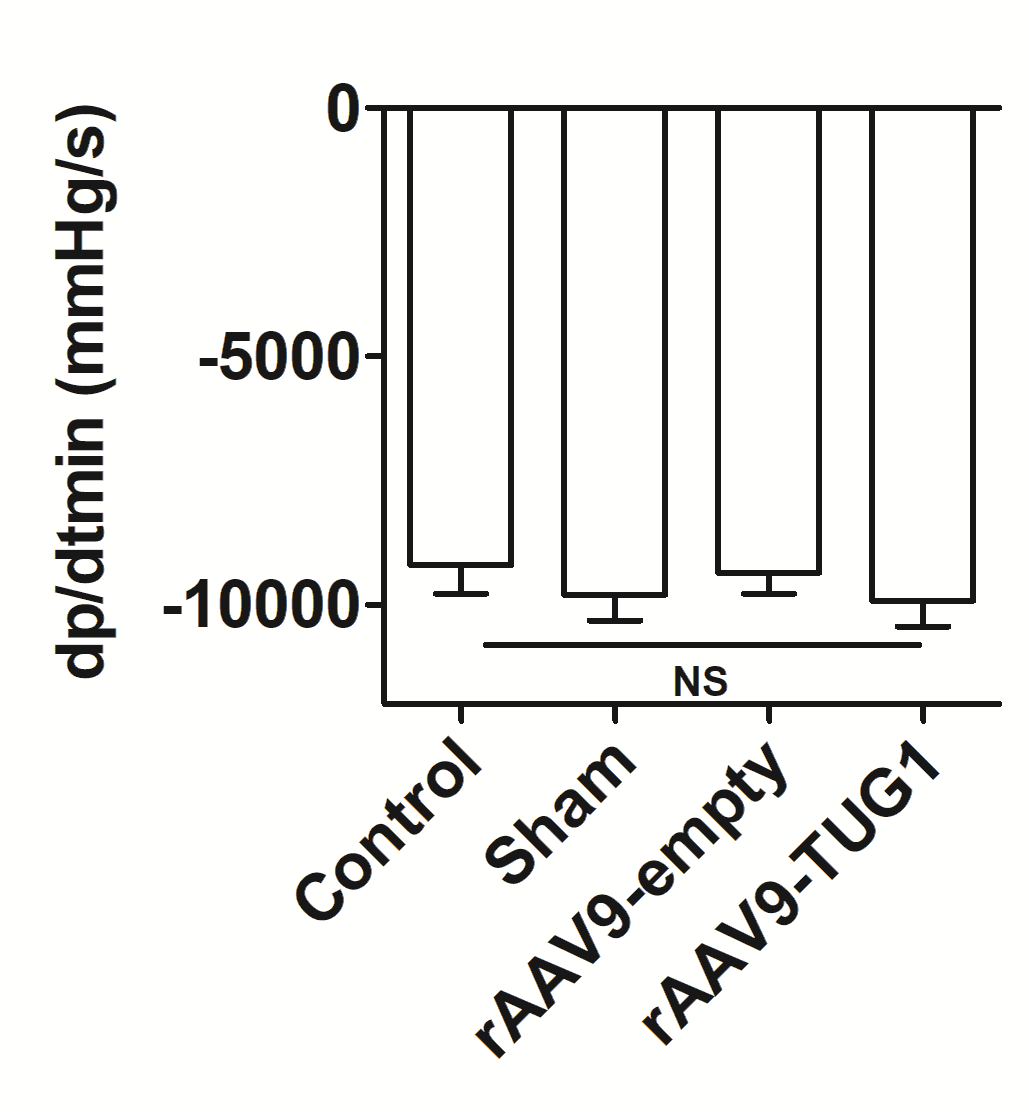
**D E**


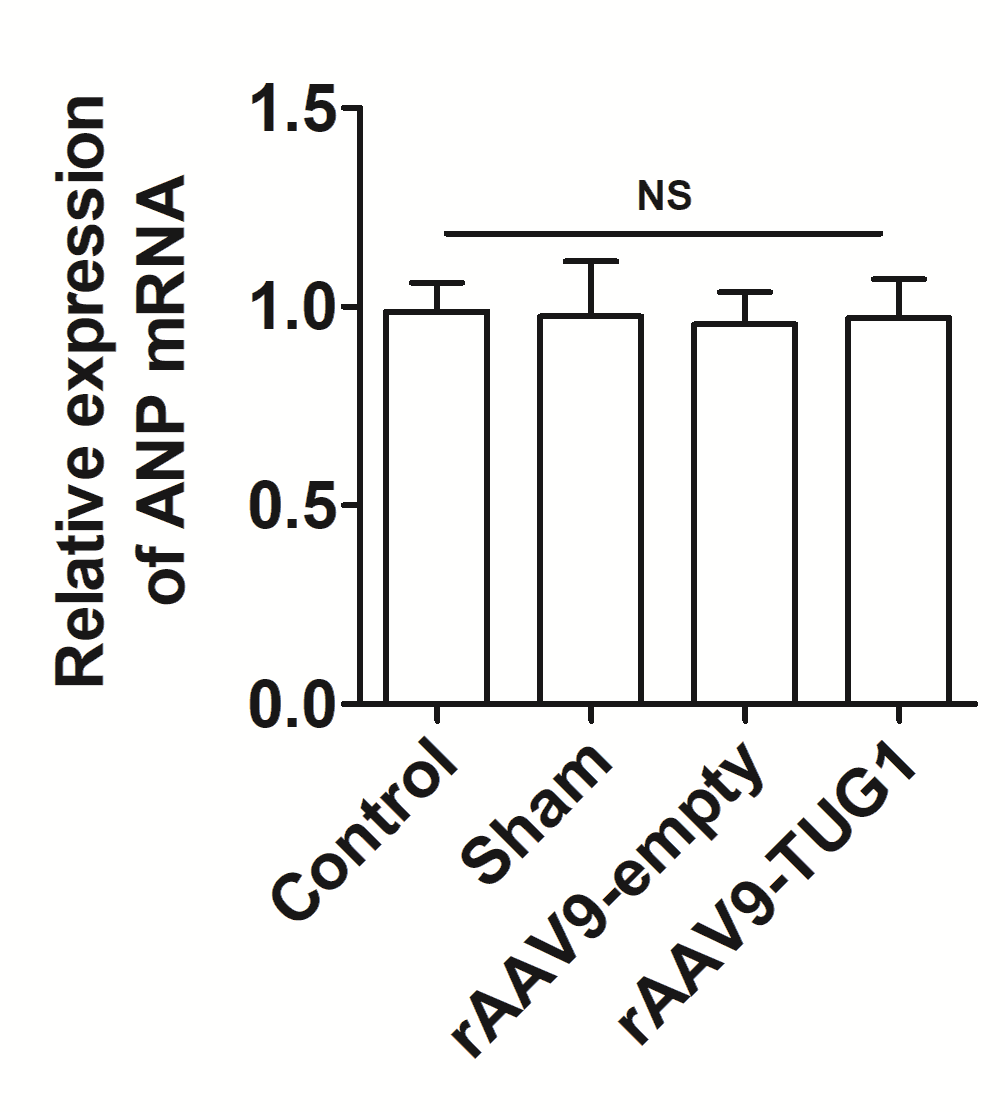

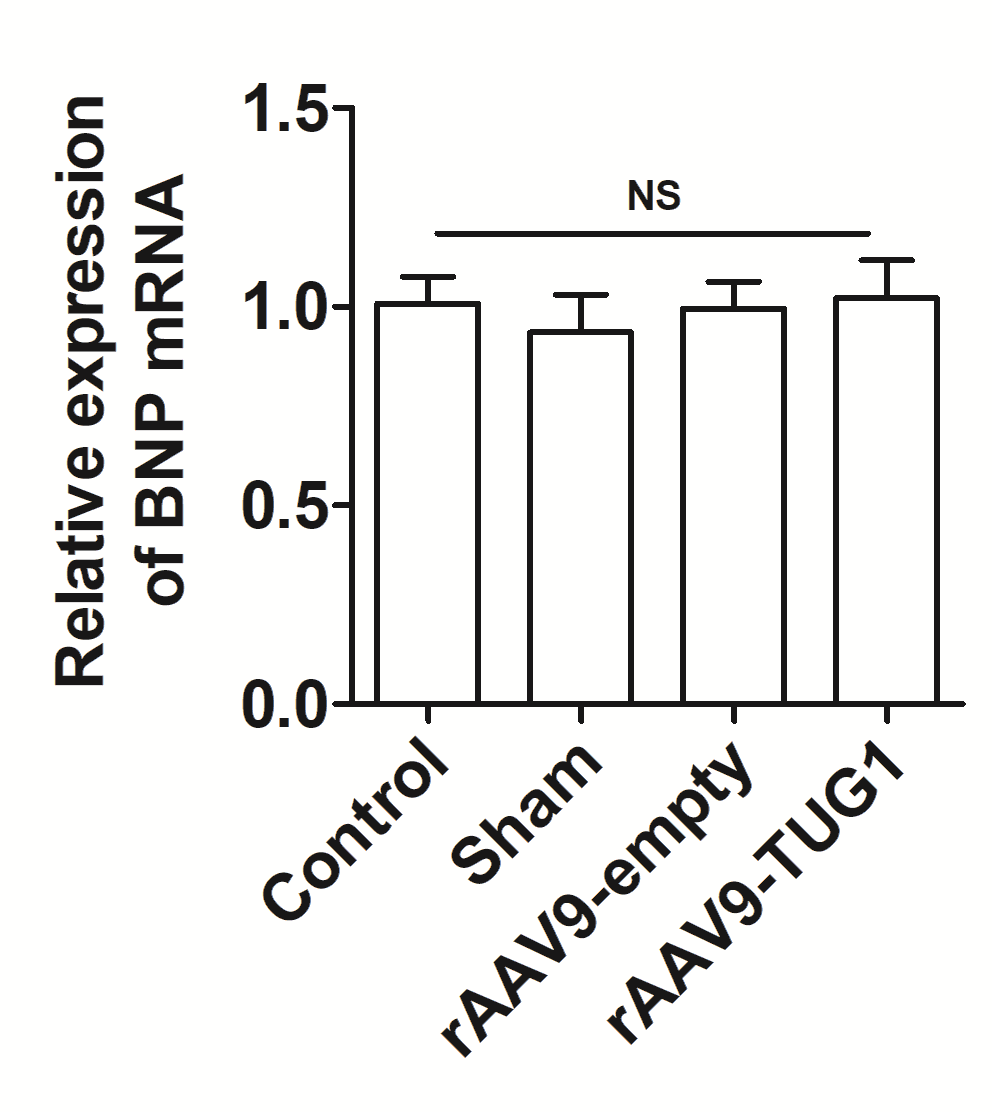

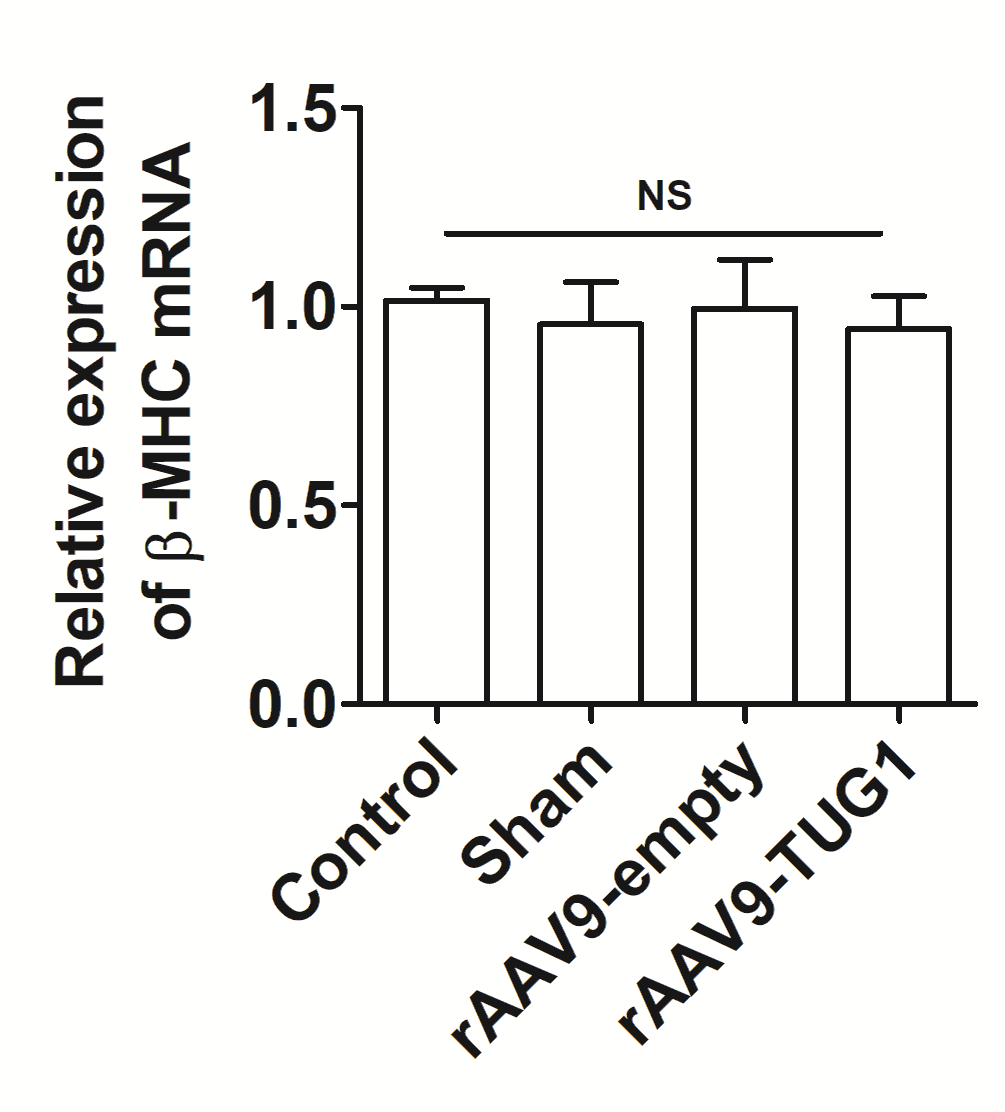
**F**

**Fig.S3**

**A:** The fluorescence intensity of GFP in the hearts of mice treated by rAAV9-TUG1-GFP. **B:** The ratio of heart weight to body weight (HW/DW) of mice with rAAV9-TUG1. (n=6, NS=*P* > 0.05 *vs* Control group). **C-D:** Echocardiography analysis of cardiac function in the mice treated with rAAV9-TUG1. (n=6, NS=*P* > 0.05 *vs* Control group; LVPW, d: left ventricle posterior wall thickness at diastole; LVPW, s: left ventricle posterior thickness at systole; LVEF, left ventricular ejection fraction). **E:** Hemodynamic parameters measured by Millar cardiac catheter system in the mice treated with rAAV9-TUG1. (n=6, NS=*P* > 0.05 *vs* Control group; dp/dtmax: peak instantaneous rate of left ventricular pressure increase; dp/dtmin: peak instantaneous rate of left ventricular pressure decline). **F:** Real-time PCR analyses for the relative mRNA levels of hypertrophic biomarkers in the heart of mice cardiomyocytes treated with rAAV9-TUG1. (n=6, NS=*P* > 0.05 *vs* Control group)**.**
